# Green-Light-Activatable Penicillin for Light-Dependent Spatial Control of Bacterial Growth, Biofilm Formation, and *In Vivo* Infection Treatment

**DOI:** 10.1021/acscentsci.5c00437

**Published:** 2025-06-11

**Authors:** Albert Marten Schulte, Jorrit W. A. Schoenmakers, Marleen van Oosten, Paul C. Jutte, Jan Maarten van Dijl, Wiktor Szymanski, Ben L. Feringa

**Affiliations:** † Centre for Systems Chemistry, Stratingh Institute for Chemistry, Faculty for Science and Engineering, University of Groningen, Nijenborgh 4, 9747 AG Groningen, The Netherlands; ‡ Department of Medicinal Chemistry, Photopharmacology and Imaging, Groningen Research Institute of Pharmacy, University of Groningen, Antonius Deusinglaan 1, 9713 AV Groningen, The Netherlands; § Department of Medical Microbiology and Infection Prevention, University of Groningen, University Medical Center Groningen, Hanzeplein 1, 9713 GZ Groningen, The Netherlands; ∥ Department of Orthopaedics, University of Groningen, University Medical Center Groningen, Hanzeplein 1, 9713 GZ Groningen, The Netherlands; ⊥ Department of Radiology, Medical Imaging Center, University Medical Center Groningen, University of Groningen, Hanzeplein 1, 9713 GZ Groningen, The Netherlands

## Abstract

Our ability to prevent, treat, and cure bacterial infections is nowadays seriously threatened by the rise of (multidrug) antimicrobial resistance (AMR), and novel molecular approaches in the antibacterial arsenal are urgently needed. To fight the development of AMR, the field of photopharmacology aims to develop photoresponsive antimicrobials allowing for noninvasive activation of the drug only at the site needed, with spatiotemporal precision, reducing the bacterial exposure to the active antimicrobial in the environment. This study reports the development and application for the first time of a green-light-activatable variant of penicillin (**Penicillin-PPG**), designed through the incorporation of a photocleavable protecting group. Here, we demonstrate that **Penicillin-PPG** shows no antimicrobial activity in the dark, while it can be precisely activated through irradiation with green light. Furthermore, we show **Penicillin-PPG**’s utility to spatially control bacterial growth, achieve light-dependent inhibition of biofilm formation, and showcase the unprecedented usage of a photoactivatable antimicrobial *in vivo* in a small animal infection model. Furthermore, we apply **Penicillin-PPG** in combination with a λ-orthogonally photocaged bioactive compound to achieve photocontrol over antimicrobial activity dependent on two distinct colors of light.

## Graphical abstract

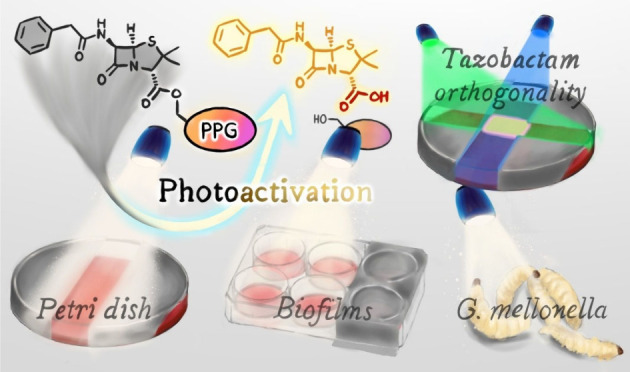

## Introduction

The discovery of penicillin by Sir Alexander Fleming almost a century ago was a major breakthrough in the treatment of bacterial infections, and antibiotics have since become the cornerstone in fighting infectious diseases.^1^ Moreover, modern procedures in healthcare such as complex surgery, chemotherapy, and organ transplantation have all emerged as a direct consequence of the existence of prophylactic antibiotic therapy. However, our ability to protect, treat, and cure patients from bacterial infections is nowadays seriously threatened due to the rise of (multidrug) antimicrobial resistance (AMR), which is caused by the selective evolutionary pressure on bacteria as a result of extensive mis- and overuse of antibiotics in medicine and the animal feed industry.^2^ AMR is currently responsible for >35.000 deaths per year in the United States,^3^ is one of the top 10 global public health threats facing humanity as declared by the World Health Organisation,^4^ and is projected to become the global leading cause of mortality by 2050.^5^

Improving the current antimicrobial stewardship is necessary to prevent a postantibiotic era. In this regard, developing novel molecular tools for the antibacterial arsenal is highly desirable. One exciting new approach is photopharmacology, which enables the light-triggered activation of drugs through incorporation of photocleavable protecting groups (photocages, PPGs) or molecular photoswitches into the drugs’ molecular structure.^6–10^ By applying this approach to antimicrobial drugs, their bioactivity can be precisely regulated, noninvasively and at specific regions of interest in the body, simply through light irradiation. This offers several advantages, namely: (i) achieving high spatial and temporal control of desired concentrations of the active drug above the minimum inhibitory concentration (MIC), (ii) avoiding gastro-intestinal side effects of the antimicrobial on the gut microbiome, and (iii) reducing the buildup of active antimicrobial and the subsequent emergence of AMR in the environment, because there is substantially less active drug secreted from the body as the antimicrobial is only released at the site of infection.

The two photochemical strategies that are utilized in photopharmacology rely on the use of PPGs and photoswitches, both featuring their own advantages and disadvantages. The use of photoswitches has the advantage of reversibility: the light-activated antimicrobial can potentially turn itself off in a photochemical or thermal back-switching process. However, the significant benefit offered by photocaged (PPG-protected) antibiotics is that they release the native antimicrobial after irradiation, restoring its inherent biological activity.^11,12^ In contrast, the introduction of a photoswitchable moiety into the molecular structure of an antimicrobial will almost always result in a loss of activity compared to the parent antimicrobial.^7–10^ Furthermore, covalently introducing a PPG in the structure of an antimicrobial often significantly reduces the biological activity of the drug in the ‘dark state’, for example, by blocking the interaction of a crucial moiety with the target enzyme. Using photoswitches, achieving a large difference in activity between ‘light’ and ‘dark’ states can be significantly more challenging, because here irradiation induces the isomerisation between two isomers, a relatively small change in chemical structure upon irradiation compared to the irradiation-dependent release of a covalently attached PPG.

While there are numerous examples of photoresponsive antimicrobials that were developed through the introduction of photoswitches into their chemical structure,^6,13–19^ achieving the same goal through the use of PPGs is relatively underexplored. A few studies have been reported, but they rely on UV to blue light irradiation,^20–24^ utilizing high energy photons that do not penetrate far into tissue, with only one recent report on a red-shifted photocleavable antimicrobial.^25^

This study set out to develop a novel photocleavable antimicrobial and demonstrate its utility to spatially control bacterial growth, achieve light-dependent inhibition of biofilm formation, and showcase its first usage *in vivo* in a small animal model. Accomplishing this required a photocleavable antimicrobial with good water-solubility and a wavelength of activation extending beyond blue light, because increasing the PPG activation wavelength from UV/blue to green light lowers the intervening tissue absorption by approximately 10-fold.^26^ As the target antimicrobial, we decided to use one of the hallmark antibiotics, penicillin, from the most widely used class of β-lactam antibiotics.^27^

## Results and Discussion

### Design and Synthesis of **Penicillin-PPG**

Penicillin’s antimicrobial activity originates from the inhibition of transpeptidases, enzymes involved in cross-linking the bacterial cell wall.^28^ Through mimicking the C-terminal alanine residues of its substrate peptide, penicillin binds and forms a covalent adduct with this target enzyme, rendering it inactive and preventing cell wall cross-linking.^28,29^ With the carboxylic acid moiety being crucial for mimicking the C-terminal substrate peptide (Figure 1), we envisioned that covalently installing a photocage at this moiety through esterification (Figure 1) would likely significantly lower the target-recognition and therefore antimicrobial activity of the Penicillin-PPG conjugate. We hypothesized that the native activity of penicillin could then be restored through light irradiation and payload release.

**Figure 1:**
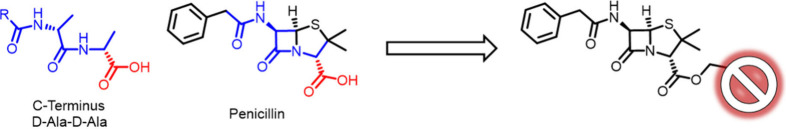
Design of a photocleavable penicillin analogue. Left: A schematic representation of the similarity between the C-terminal substrate peptide of transpeptidase and penicillin, highlighting the common carboxylic acid moiety (red). Right: The proposed design of a photocleavable penicillin analogue through blockage of the carboxylic acid moiety with a photocleavable protecting group.

However, while blocking the carboxylic acid moiety of penicillin would theoretically significantly reduce its antimicrobial activity, blocking this polar moiety also negatively affects the solubility of the final photocaged compound. Therefore, it requires the use of a photocage that, besides featuring activation beyond blue light, also features adequate water-solubility.

Usually, activation-wavelength and water-solubility of PPGs are inversely correlated, since red-shifting a photocage is often achieved through an extended conjugation, leading to larger, hydrophobic compounds.^30–33^ However, in 2019, Bojtár et al. developed a coumarin-based photocage with an extended conjugation, making it green-light-responsive, while simultaneously improving its aqueous solubility through the introduction a charged pyridinium moiety.^34^ We envisioned that the use of this photocage would satisfy our requirements regarding both the wavelength of activation and water solubility. The PPG precursor **1** was synthesized following the published procedure^34^ and installed on the carboxylic acid moiety of penicillin through a Steglich esterification to yield compound **2** (Figure 2a). Subsequent methylation of the pyridine moiety yielded the final compound **Penicillin-PPG** (see the SI for full characterization).

**Figure 2:**
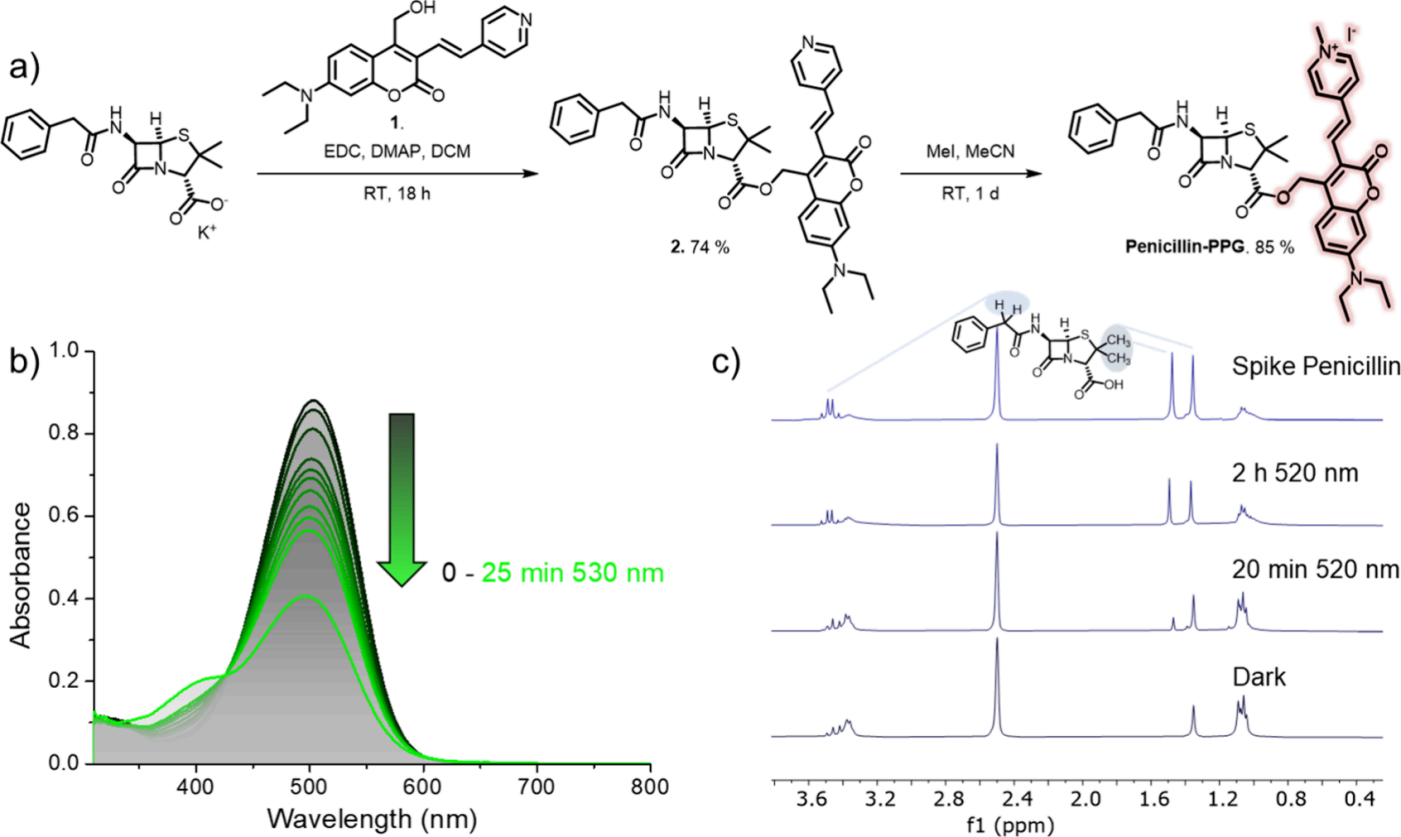
Synthesis and photochemical characterization of **Penicillin-PPG**. (a) Synthetic scheme for the formation of **Penicillin-PPG**. (b) UV–vis absorption spectra of samples of **Penicillin-PPG** (20 μM, LB with 1% DMSO, 37 °C), before and after irradiation (λ = 530 nm, black and green lines, respectively, total irradiation time 25 min). (c) ^1^H NMR spectra of samples of **Penicillin-PPG** (2 mM, DMSO-*d*_6_/D_2_O 1:1) in the dark and after irradiation with green light (λ = 520 nm). The release of penicillin was confirmed through spiking with a penicillin-potassium salt (top spectrum).

UV–vis spectroscopy of **Penicillin-PPG** in Lysogeny Broth (LB) showed that the compound responded to green light irradiation (Figure 2b, λ = 530 nm), while ^1^H NMR (Figure 2c and SI Section 2.2) analysis of irradiated samples revealed that upon green light irradiation penicillin was liberated from **Penicillin-PPG**.

### Light-Dependent Antimicrobial Activity in Liquid Cultures Using **Penicillin-PPG**

To study the light-dependent antimicrobial activity of **Penicillin-PPG**, the penicillin-susceptible *Escherichia coli* DH5α strain was used. Initially, the MIC-value of penicillin against this strain was determined to be 75 μM (SI Section 3.1). Solutions of **Penicillin-PPG** were prepared in LB and were either kept in the dark or irradiated with green light (λ = 530 nm) for 1 h. Subsequently, the samples were inoculated with *E. coli*, and optical density (OD) growth curves were recorded for 24 h at 37 °C. The irradiated **Penicillin-PPG** samples effectively prevented the growth of *E. coli* DH5α (Figure 3a, green line), whereas the samples that were kept in the dark (Figure 3a, black line) resulted in bacterial growth curves similar to the controls in the absence of penicillin (Figure 3a, red and blue lines). These results illustrate that while **Penicillin-PPG** showcases the desired minimal activity in the dark, the antimicrobial activity could be effectively restored through green-light irradiation.

**Figure 3:**
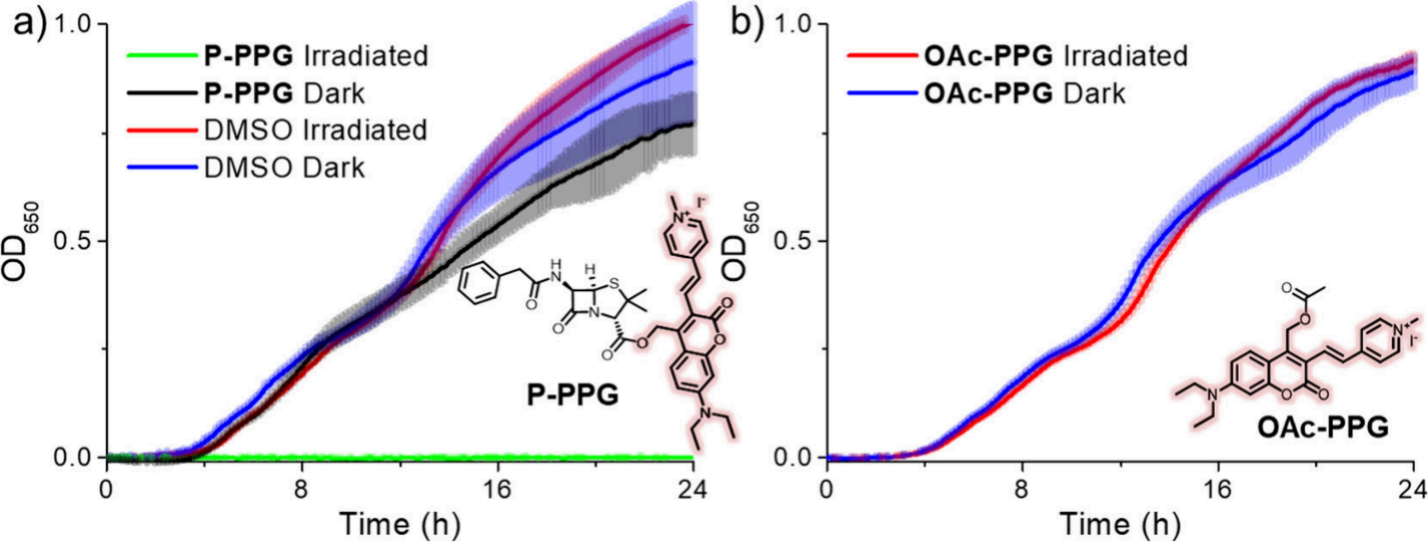
Growth of *E. coli* in LB with or without green light-irradiated **Penicillin-PPG**. Growth curves of *E. coli* DH5α were determined in triplicate, and shown are the averages and standard deviations. 80 μM **Penicillin-PPG** (a) or **OAc-PPG** (b) was used. The final concentration of DMSO was 3%. Cells were grown overnight at 37 °C.

To study whether the observed light-dependent antimicrobial effect was caused by the release of penicillin itself and not the release of the potentially toxic photoproducts from PPG, an additional experiment was performed using the same PPG bearing acetic acid as a model payload without antimicrobial activity (Figure 3b, **OAc-PPG**). This PPG releases the same photoproducts as **Penicillin-PPG**, with the crucial exception of the payload penicillin, which is replaced by acetic acid. Irradiation of **OAc-PPG** had no effect on the normal growth of *E. coli* DH5α (Figure 3b), regardless of whether the samples were irradiated or kept dark. This experiment showcases that the photoproducts stemming from the PPG itself are noncytotoxic toward the *E. coli* strain used, and that the green light induced antimicrobial effect observed in the case of **Penicillin-PPG** stems from the light-controlled release of penicillin.

### Light-Dependent Spatial Control of Bacterial Growth Using **Penicillin-PPG**

Encouraged by the results obtained in liquid cultures, we set out to study whether control over bacterial growth could be achieved through site-specific light irradiation of **Penicillin-PPG** with spatial precision. An experiment was set up in which agar plates were poured containing **Penicillin-PPG** at a concentration slightly above the MIC of penicillin. The plates were inoculated with *E. coli* DH5α, and part of the plate was covered from light using an aluminum sticker. The other part was irradiated for 1 h with green light (Figure 4a), and the plates were incubated at 37 °C overnight.

**Figure 4:**
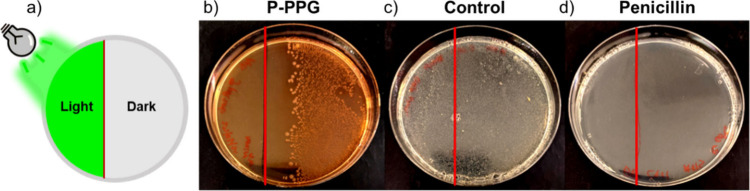
Spatial control of bacterial growth with **Penicillin-PPG**. (a) Schematic representation of the experimental setup, in which part of an agar plate inoculated with *E. coli* DH5α was covered with an aluminum sticker (‘dark’) while the other part was irradiated (‘light’, λ = 530 nm, 1 h). (b) Experimental results of agar plates containing **Penicillin-PPG**, (c) DMSO control, or (d) penicillin (90 μM **Penicillin-PPG** or penicillin, 3% DMSO in LB-agar). Of note, the color of the plate containing **Penicillin-PPG** appears slightly red, which is caused by the dissolved **Penicillin-PPG**.

Irradiation of the plate containing **Penicillin-PPG** effectively prevented bacterial growth on the irradiated part (Figure 4b), while the dark part of the plate presented bacterial colony formation. These results demonstrate that in solid medium spatial control over penicillin release can be achieved through green light irradiation. Inhibition of bacterial growth by light-activated **Penicillin-PPG** was observed even slightly beyond the irradiated part. This effect could be caused by diffusion of uncaged penicillin through the agar medium. In addition, it might be enhanced by activation of the **Penicillin-PPG** due to incomplete shielding by the aluminum sticker or reflection of the green light through the agar medium. Control experiments in which no **Penicillin-PPG** was present in the agar illustrated that green light irradiation itself was nontoxic to the bacteria, because both the irradiated and dark parts of the blank plate presented bacterial colony formation (Figure 4c), and green-light irradiation itself had no effect on the activity of penicillin, because bacterial growth was inhibited both on the irradiated and dark parts of a control plate containing penicillin (Figure 4d).

### Prevention of Biofilm Formation Using **Penicillin-PPG**

To test whether **Penicillin-PPG** can also be applied for the prevention of biofilm formation, we performed an experiment with the *Staphylococcus epidermidis* American Type Culture Collection (ATCC) strain 35984, which is known to be a good biofilm former.^35^ The MIC-value of this strain for penicillin was determined to be 32 μM (SI Section 3.4). Incubation of *S. epidermidis* in growth medium with a sterile 16 mm glass coverslip resulted in biofilm formation on the coverslip within 24 h at 37 °C, irrespective of whether the culture was irradiated with green light (λ = 530 nm) for 2 h or incubated in the dark. This biofilm formation was visualized by Crystal Violet staining (Figure 5a,b) and quantified by determining the bacterial colony-forming units (CFUs) upon sonication of the coverslips and subsequent plating (SI Section 3.4). In contrast, no biofilm formation on the coverslips was detectable within 24 h when *S. epidermidis* was incubated in growth medium with penicillin (slightly above MIC concentration), neither visually (Figure 5c,d) nor by CFU counting, regardless of green light irradiation or dark conditions. Interestingly, irradiation of *S. epidermidis* with green light in the presence of **Penicillin-PPG** (slightly above the MIC concentration) resulted in clearly reduced biofilm formation on the coverslips (Figure 5f) compared to the coverslips that were kept in the dark (Figure 5e). Moreover, CFU counting showed that irradiation of **Penicillin-PPG** with green light led to an almost 2-log (97%) reduction in the biofilm-embedded bacterial load on the coverslips compared to the dark conditions (SI Section 3.4). Furthermore, the bacterial load on the coverslips upon incubation in the presence of **Penicillin-PPG** in the dark was comparable to that on the coverslips incubated in the absence of antibiotics in the dark with or without green-light exposure. This shows that **Penicillin-PPG** prevents *S. epidermidis* biofilm formation only upon activation with green light.

**Figure 5:**
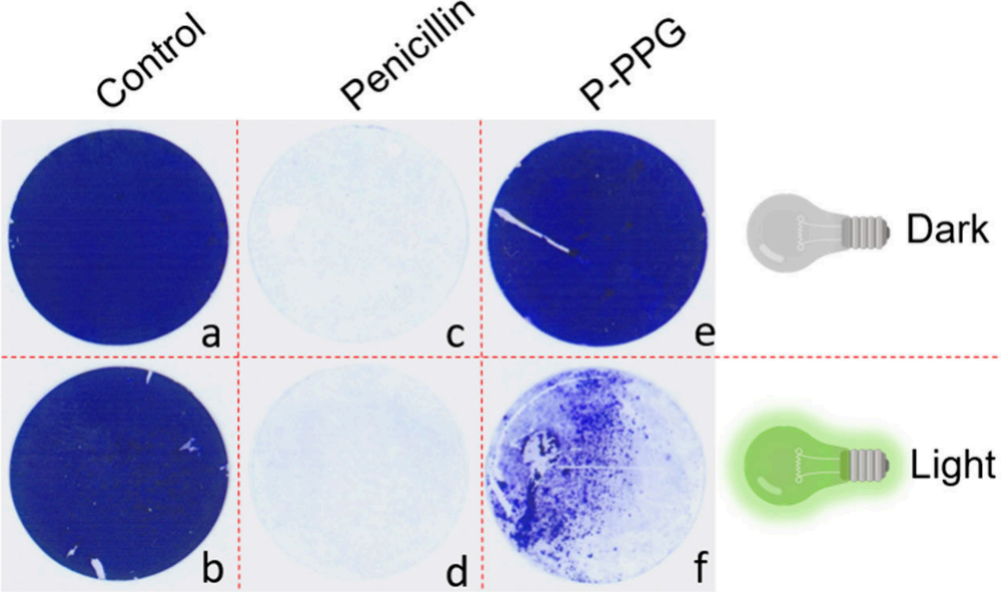
Prevention of *S. epidermidis* biofilm formation by green light-activated **Penicillin-PPG**. *S. epidermidis* ATCC 35984 was used to inoculate growth medium in microtiter plates supplemented with **Penicillin-PPG**, penicillin or without supplementation (DMSO control). After irradiation for 2 h with green light or incubation in the dark, biofilms were grown on coverslips for 24 h at 37 °C. Subsequently, the coverslips were stained with Crystal Violet to detect biofilm formation. (a, b) Biofilm growth in the absence of antibiotic, regardless of irradiation with green light. (c, d) Absence of biofilm formation in the presence of penicillin (52 μM), regardless of irradiation with green light. (e) Unimpaired biofilm formation in the presence of **Penicillin-PPG** (52 μM) upon incubation in the dark. (f) Impaired biofilm formation in the presence of **Penicillin-PPG** after irradiation with green light.

### *In Vivo* Treatment of Bacterial Infection with Green-Light-Activated **Penicillin-PPG**

To assess the feasibility of using **Penicillin-PPG** for *in vivo* treatment of infection, we used a small animal infection model that is based on larvae of the greater wax moth *Galleria mellonella*. This model was chosen because the innate immune defenses of these larvae resemble those of humans,^36^ and because it was previously successfully used in antimicrobial photodynamic therapy (aPDT) studies.^37^ For infecting the larvae, we applied the *S. aureus* strain SH1000, which is highly susceptible to penicillin with a MIC of 0.2 μM (SI Section 3.5). First, it was established that injection of 0.5 × 10^6^ CFUs of exponentially growing *S. aureus* SH1000 killed approximately 50% of the larvae within 72 h and ∼66% of the larvae in 96 h. Then, to check for possible confounding effects on larval survival, the following control experiments were performed: (1) uninfected larvae were subjected to 2 h of irradiation with green light, (2) uninfected larvae were injected with phosphate-buffered saline (PBS) to assess possible effects of the injection trauma, and (3) uninfected larvae were injected with **Penicillin-PPG** at twice the MIC-value to assess the toxicity of **Penicillin-PPG**, with or without prior green light (λ = 530 nm) irradiation for 2 h. After 0, 24, 48, 72, and 96 h of follow up, none of the these potentially confounding procedures had any significant influence on the larval survival (Figure 6a; Gehan–Breslow–Wilxocon test χ^2^ = 2.01, *P*-value = 0.16).

**Figure 6:**
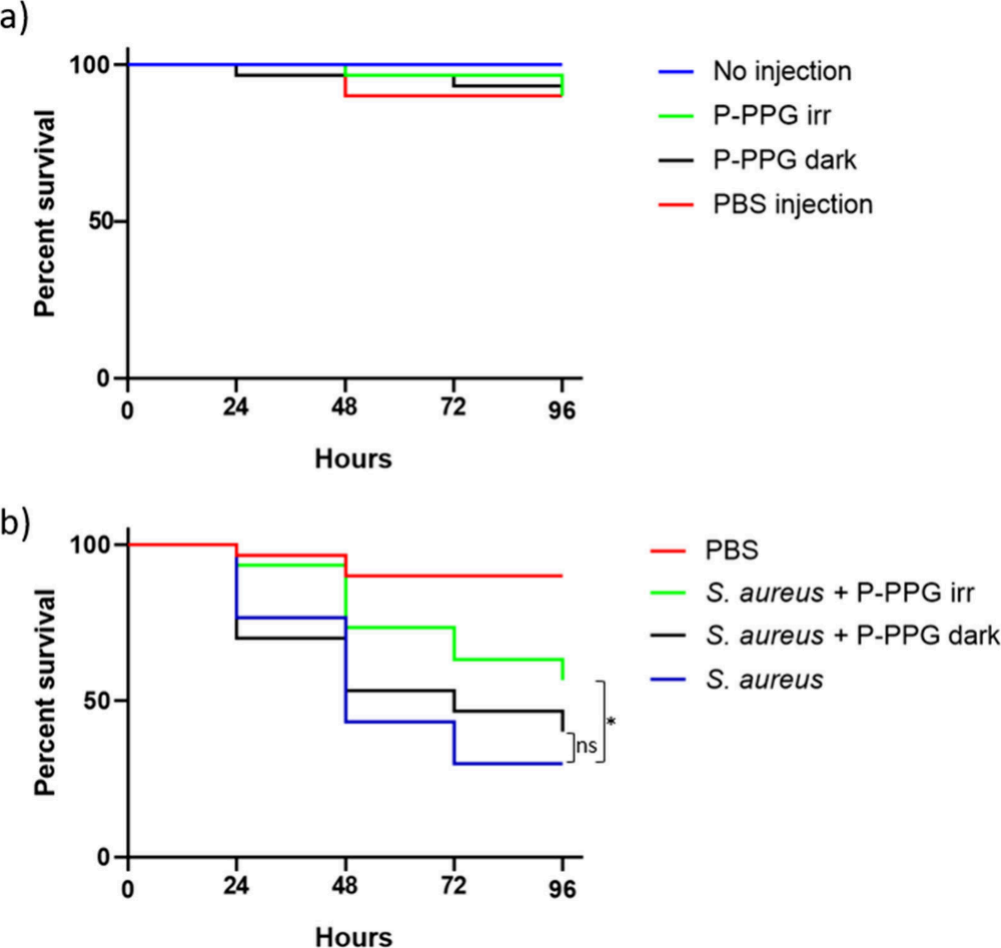
Kaplan–Meier survival curves of *G. mellonella* after *S. aureus* infection and treatment with **Penicillin-PPG**. *N* = 30 larvae per group at *t* = 0. (a) There are no significant differences in the survival of uninfected larvae that received (1) no injection, (2) **Penicillin-PPG** injection and subsequent 2 h irradiation with green light, (3) **Penicillin-PPG** injection and subsequent incubation in the dark, and (4) PBS injection. (b) Compared to *S. aureus*-infected larvae that received no treatment (blue line), *S. aureus*-infected larvae that were injected with **Penicillin-PPG** and received green light irradiation for 2 h (green line) showed a significantly increased survival. This was not the case for *S. aureus*-infected larvae treated with **Penicillin-PPG** that were kept in the dark (black line). *P < 0.05; ns, not significant.

Next, to determine the efficacy of **Penicillin-PPG** treatment *in vivo*, the larvae were injected with 0.5 × 10^6^ CFUs of exponentially growing *S. aureus* SH1000, with or without **Penicillin-PPG** at twice the MIC-value of penicillin. Upon injection, half of the larvae were immediately subjected to 2 h of green light irradiation, whereas the other half was kept in the dark. After 2 h, all larvae were placed in a dark incubator, and their survival was monitored at 24, 48, 72, and 96 h postirradiation. As shown in Figure 6b, injection with *S. aureus* alone killed approximately 70% of the larvae after 96 h. In contrast, only ∼40% of the infected larvae that were simultaneously injected with **Penicillin-PPG** and irradiated for 2 h with green light were killed by the *S. aureus* infection at this time point, respectively. Excitingly, Kaplan–Meier analysis showed a significantly better survival of *S. aureus*-infected larvae that were injected with **Penicillin-PPG** and received treatment with green light (green line) compared to *S. aureus*-infected larvae who were not injected with **Penicillin-PPG** (blue line) (Gehan–Breslow–Wilcoxon test χ^2^ = 6.44, *P* = 0.011). Furthermore, there was no significant difference in survival between *S. aureus*-infected larvae that received **Penicillin-PPG** but were kept in the dark (black line) and *S. aureus*-infected larvae that were not injected with **Penicillin-PPG** (blue line) (χ^2^ = 0.18, *P* = 0.67), indicating that indeed irradiation was required to achieve the antimicrobial effect. However, although the Kaplan–Meier curves suggest that survival among infected larvae that received **Penicillin-PPG** and were irradiated (green line) is higher compared to larvae that were kept in the dark (black line), this difference is not statistically significant (χ^2^ = 3.33, *P* = 0.067). A two-way ANOVA with a subsequent Sidak multiple comparisons test showed that this was the case at all measured time points (24 h, *P* = 0.16; 48 h, *P* = 0.23; 72 h, *P* = 0.33; 96 h, *P* = 0.33). In summary, treatment with **Penicillin-PPG** and irradiation with green light resulted in significantly higher larval survival than that in the control group. While irradiation was required to produce significantly higher survival, the difference in survival between the irradiated and nonirradiated groups themselves was not found to be statistically significant (*P* = 0.067).

### Toward Three-Dimensional Control over Antimicrobial Activity

When a single color of light is required to achieve a biological effect, using a laser to irradiate a model will result in a two-dimensional resolution since all of the structure hit by the irradiation beam will be affected, unless multiphoton absorption processes are involved where the focal point of the laser can give rise to three-dimensional resolution. Contrastingly, if activation of the effect would require irradiation with two colors of light, only where the two irradiation beams would overlap would the function be activated, increasing the spatial resolution of control over said function. Ultimately, if the beams would be used in an orthogonal set up with the two colors of light coming from different directions, in theory three-dimensional control over the function could be achieved using this dual-color system. Similar strategies have already been explored successfully in materials science, where high resolutions could be obtained (20–50 μm).^38^ However, in the latter example, photoactivation was applied to initiate polymerization. Here it should be noted that the resolution may be lower in processes that involve the diffusion of activated compounds.

The strains of bacteria used so far in this manuscript are penicillin-sensitive, and therefore, the release of penicillin alone suffices to result in the antimicrobial effect. To evaluate the validity of our dual-color concept, we next opted to use a penicillin-resistant strain. Here we chose a variant of the *E. coli* DH5α strain that features a pET-Duet vector encoding a β-lactamase enzyme. These enzymes hydrolyze the β-lactam ring of certain β-lactam antimicrobials, rendering bacteria that produce them resistant to these antibiotics.^39^ β-Lactamases can be counteracted by β-lactamase inhibitors (BLIs) such as tazobactam, resensitizing resistant bacteria to β-lactam antibiotics. Therefore, achieving an antimicrobial effect on the penicillin-resistant strain would rely on the combined use of both penicillin and a BLI, forming the basis for the 3D control described above.

To achieve the dual-color dependent activation, we sought a PPG that would respond to a wavelength of light at which **Penicillin-PPG** remains largely intact (Figure 7). The absorption spectrum of **Penicillin-PPG** features a main absorption band around 500 nm and minimal absorption in the 390–400 nm spectral region (Figure 2b). This latter region is the part of the visible spectrum where the main absorption band of an unmodified diethylaminocoumarin (DEAC) PPG is located (see Figure 8, *vide infra*). Therefore, we envisioned that installing a DEAC PPG on the BLI tazobactam could allow for the selective release of tazobactam upon irradiation with violet light (∼390–400 nm), without activating **Penicillin-PPG** to a significant degree. **Tazobactam-PPG** was thus designed, featuring a diethylamino-coumarin PPG installed at the carboxylic acid moiety (Figure 7a). Furthermore, the design of **Tazobactam-PPG** also featured two 2-hydroxyethylene moieties at the aniline, which were included to increase the aqueous solubility of the compound.

**Figure 7:**
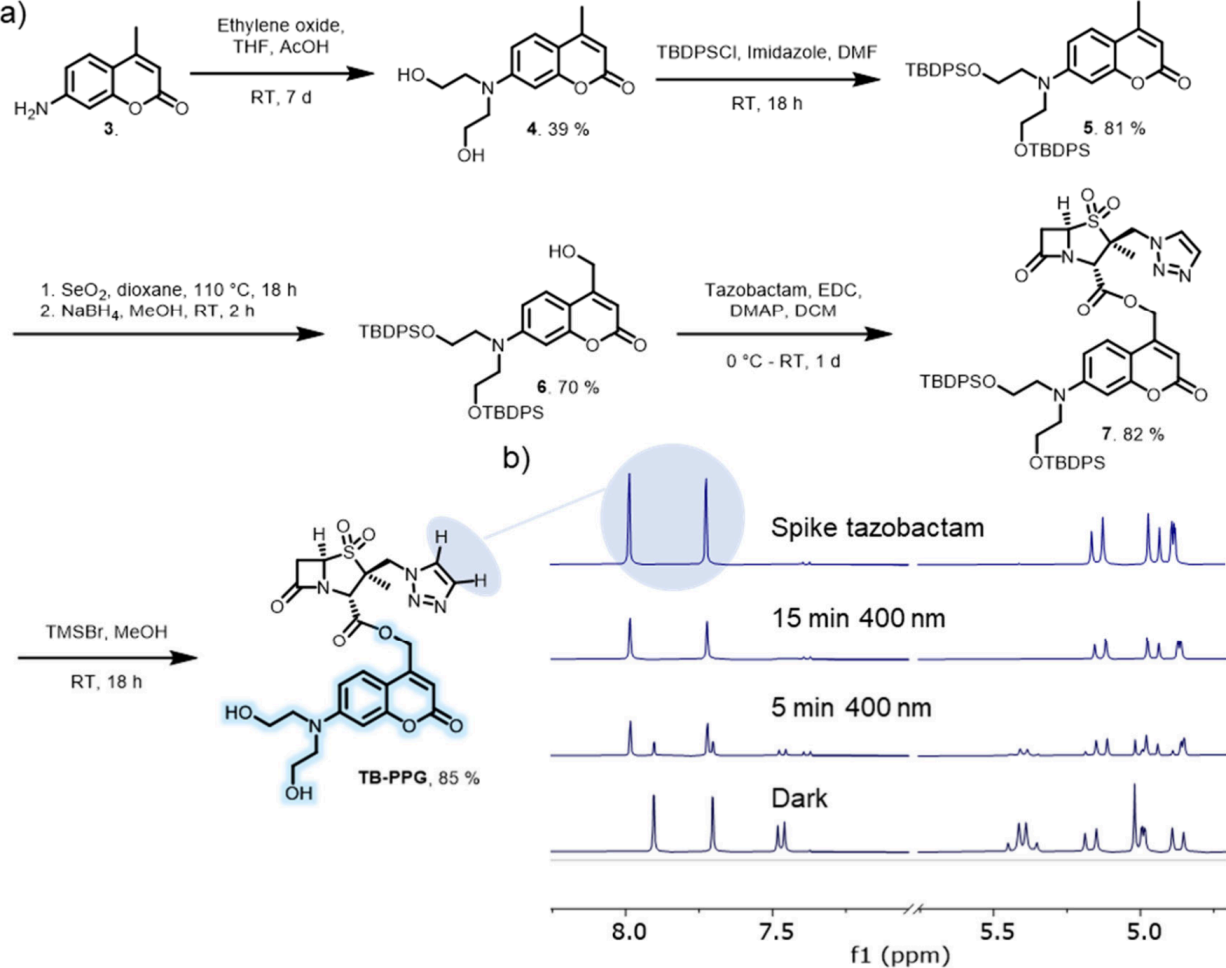
(a) Synthetic scheme for the preparation of **Tazobactam-PPG**. (b) Partial ^1^H NMR spectra of samples of **Tazobactam-PPG** (2 mM, DMSO-*d*_6_/D_2_O 1:1) in the dark and after irradiation with violet light (λ = 400 nm) for the times indicated. The release of tazobactam was confirmed through a spike with tazobactam (top spectrum).

**Figure 8:**
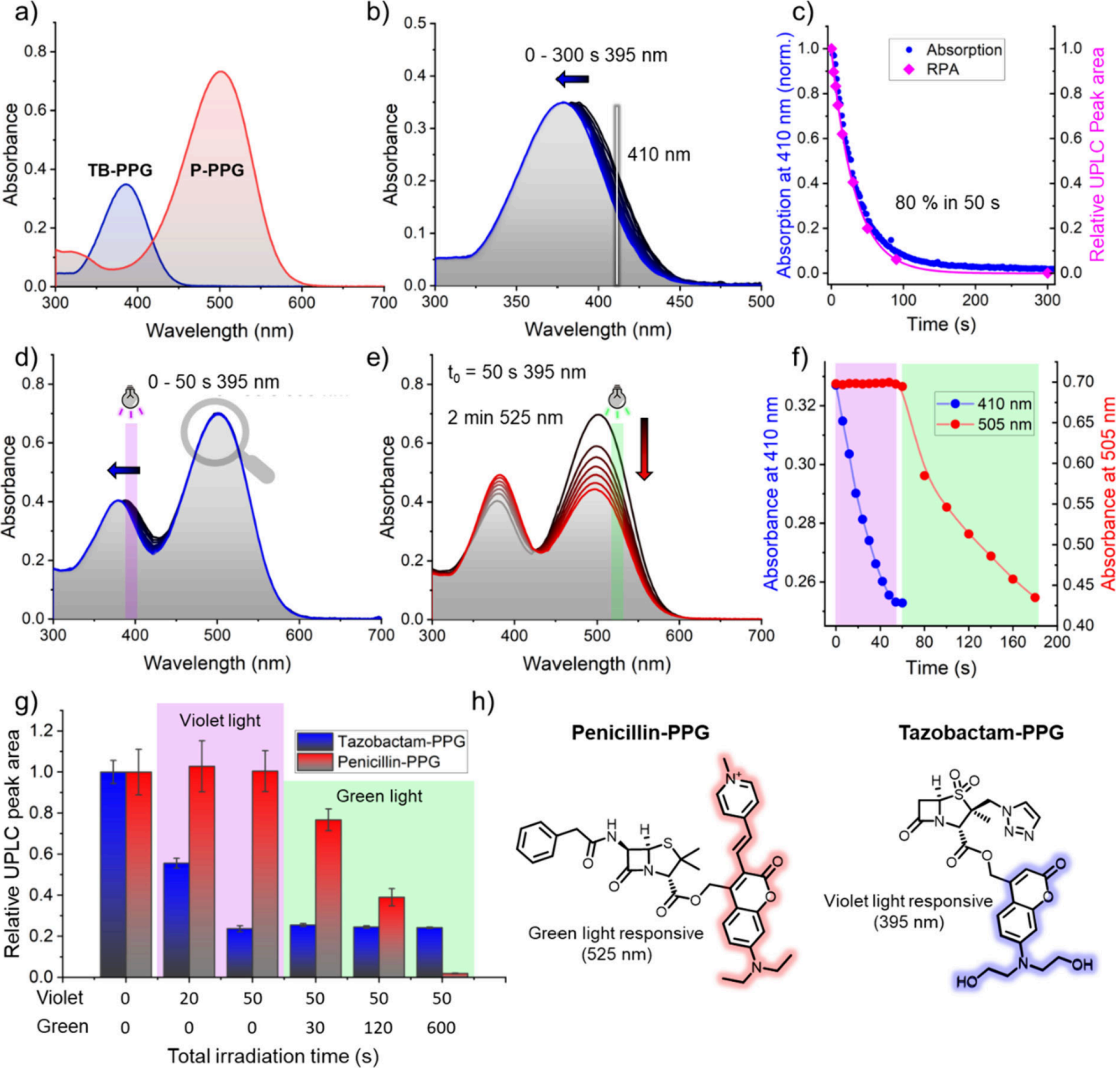
(a) Overlay of UV–vis absorption spectra of **Tazobactam-PPG** (blue) and **Penicillin-PPG** (red), both at 20 μM in water, 0.5% DMSO, 37 °C. (b) UV–vis absorption spectra of a sample of **Tazobactam-PPG** (20 μM, water, 0.5% DMSO, 37 °C) before and during irradiation (λ = 395 nm, black and blue lines, respectively, total irradiation time 5 min). (c) Time-dependent, normalized absorption values at 410 nm of the absorption spectra shown in (b) (blue curve) as well as relative UPLC peak area (RPA) of the peak corresponding to **Tazobactam-PPG** in UPLC-MS chromatograms upon increasing irradiation time (pink curve). (d) Absorption spectra of a mixture of **Tazobactam-PPG** and **Penicillin-PPG** (both 20 μM, water, 1% DMSO, 37 °C) in the dark (black line) and during irradiation with violet light (λ = 395 nm, 50 s, blue line). (e) Absorption spectra of the mixture of **Tazobactam-PPG** and **Penicillin-PPG** shown in (d) after irradiation with violet light for 50 s (black line) followed by irradiation with green light (λ = 525 nm, 2 min, red lines). (f) Time-dependent absorption values at 410 and 505 nm of the absorption spectra shown in parts (d) and (e), showing the effect of violet light irradiation (first 50 s, violet box) and green light irradiation (after 50 s, green box). (g) Relative peak areas based on integration of the UPLC-MS peaks corresponding to **Tazobactam-PPG** and **Penicillin-PPG** after irradiation of a mixture of the two compounds (both 20 μM, water, 1% DMSO), with violet or green light for the indicated irradiation times. (h) Structures of **Tazobactam-PPG** and **Penicillin-PPG**.

The synthesis of **Tazobactam-PPG** started from commercially available 7-amino-4-methylcoumarin **3** (Figure 7a). The hydroxyethylene functionality was installed on the aniline moiety by using a nucleophilic ring-opening of ethylene oxide, yielding compound **4**. Subsequently, the primary alcohol moieties were protected with a silyl protecting group, forming compound **5**. The benzylic position was oxidized using selenium dioxide, and the resulting crude aldehyde was reduced to the primary alcohol using NaBH_4_, yielding compound **6**. Primary alcohol **6** was esterified with tazobactam in a Steglich-esterification reaction, yielding compound **7**. Finally, deprotection of the silyl moieties required mild conditions regarding the highly sensitive β-lactam ring of compound **7**. Reaction with a catalytic amount of TMS-bromide in methanol^40^ provided the final compound **Tazobactam-PPG**. ^1^H NMR spectroscopy of **Tazobactam-PPG** in a D_2_O/DMSO-*d*_6_ mixture revealed that upon irradiation with violet light (λ_max_ = 400 nm), tazobactam was indeed liberated from **Tazobactam-PPG** (Figure 7b).

### Orthogonality of Uncaging

In a mixture containing two types of PPGs, selective activation of the red-shifted PPG (RS-PPG) can usually be achieved in a straightforward manner, given that the blue-shifted PPG (BS-PPG) simply does not absorb at the wavelength used for activation of the RS-PPG. However, the challenge in the design of wavelength-selective photouncaging systems is to selectively uncage the BS-PPG without significantly uncaging the RS-PPG.^41^ This issue arises from the fact that the RS-PPG always features some degree of absorption at the irradiation wavelength used to activate the BS-PPG.

We set out to study the selectivity of uncaging of our system by relying on **Penicillin-PPG** and **Tazobactam-PPG** using UV–vis spectroscopy. Comparison of the absorption spectrum of **Tazobactam-PPG** with that of **Penicillin-PPG** revealed that their main absorption bands are well separated; with **Tazobactam-PPG**’s absorption band located at ∼390 nm, almost exactly overlapping with the part of the spectrum where **Penicillin-PPG** displays the lowest absorbance (Figure 8a). Molar absorptivity coefficients of both compounds, determined at the irradiation wavelength of violet light (λ = 395 nm), were 3.91 × 10^3^ and 16.2 × 10^3^ M^–1^ cm^–1^ for **Penicillin-PPG** and **Tazobactam-PPG**, respectively (SI Section 2.5).

Irradiation of **Tazobactam-PPG** with violet light (λ = 395 nm) in water led to changes in its absorption spectrum, showing a clear hypsochromic shift (Figure 8b) characteristic for the formation of the coumarin-alcohol.^42–44^ Through normalization of the difference in absorption at a single wavelength, we observed that ∼80% photochemical conversion of **Tazobactam-PPG** was achieved after 50 s of irradiation with violet light (Figure 8c, SI Section 2.3). These results, based on UV–vis absorption measurements, were verified with UPLC-MS experiments. This revealed an almost identical kinetic profile for the conversion of **Tazobactam-PPG** judged by integration of its peak in the UPLC-MS chromatogram at λ = 400 nm (Figure 8c, pink curve, SI Section 2.4).

Subsequently, the orthogonality of uncaging was evaluated through mixing **Tazobactam-PPG** and **Penicillin-PPG** at equal concentrations (20 μM) in water. Irradiation of this mixture with violet light (λ = 395 nm) for 50 s (the time required to reach ∼80% uncaging of **Tazobactam-PPG**) led to significant changes in the absorption spectrum of **Tazobactam-PPG**, while not affecting the absorption spectrum of **Penicillin-PPG** (Figure 8d), indicating a high selectivity of **Tazobactam-PPG** uncaging over **Penicillin-PPG** with violet light. This effect can be observed more easily upon following the absorption spectra of both PPGs over time at a single wavelength (Figure 8f). In the initial 50 s of irradiation with violet light, the absorption at 410 nm drops rapidly, indicating uncaging of **Tazobactam-PPG**, whereas the absorption of **Penicillin-PPG** at 505 nm is minimally affected. Then, when the mixture is irradiated with green light (λ_max_ = 525 nm), the absorption of **Penicillin-PPG** at 505 nm drops (Figure 8e,f).

To further confirm the orthogonality of uncaging, the experiments shown in Figure 8d–f were followed by UPLC-MS. Integration of the peaks of **Tazobactam-PPG** and **Penicillin-PPG** at 400 and 505 nm, respectively, revealed a high degree of selectivity (Figure 8g). Upon violet light irradiation, 80% consumption of **Tazobactam-PPG** was achieved within 50 s, whereas **Penicillin-PPG** remained unaffected. Subsequently, upon green light irradiation, **Penicillin-PPG** was also consumed, whereas the integral of the **Tazobactam-PPG** peak remained unaffected.

Encouraged by these results, we set out to study the dual-color, λ-orthogonal system in a biological setting. Initially, the required concentrations of benzylpenicillin and tazobactam for growth inhibition of the β-lactam resistant strain were evaluated (SI Section 3.1). At a benzylpenicillin concentration of 80 μM, a concentration of 180 μM tazobactam was required to fully inhibit bacterial growth. A mixture containing **Penicillin-PPG** and **Tazobactam-PPG** (in short, **Mix**) slightly above these concentrations (90 and 200 μM, respectively) was prepared in LB and inoculated with an overnight culture of *E. coli* DH5α. The mixture was aliquoted in a sterile 96-well plate and kept in the dark, irradiated with violet light (1 min), green light (1 h), or both light sources. Subsequently, the cells were incubated at 37 °C overnight, and growth curves were recorded in a plate reader. Only the samples containing **Mix** that was irradiated with both violet and green light completely inhibited bacterial growth (Figure 9a, blue line). In the dark, no antimicrobial effect was observed (Figure 9a, black line), whereas irradiation of **Mix** with only green or only violet light merely delayed bacterial growth (Figure 9b, green and purple lines).

**Figure 9:**
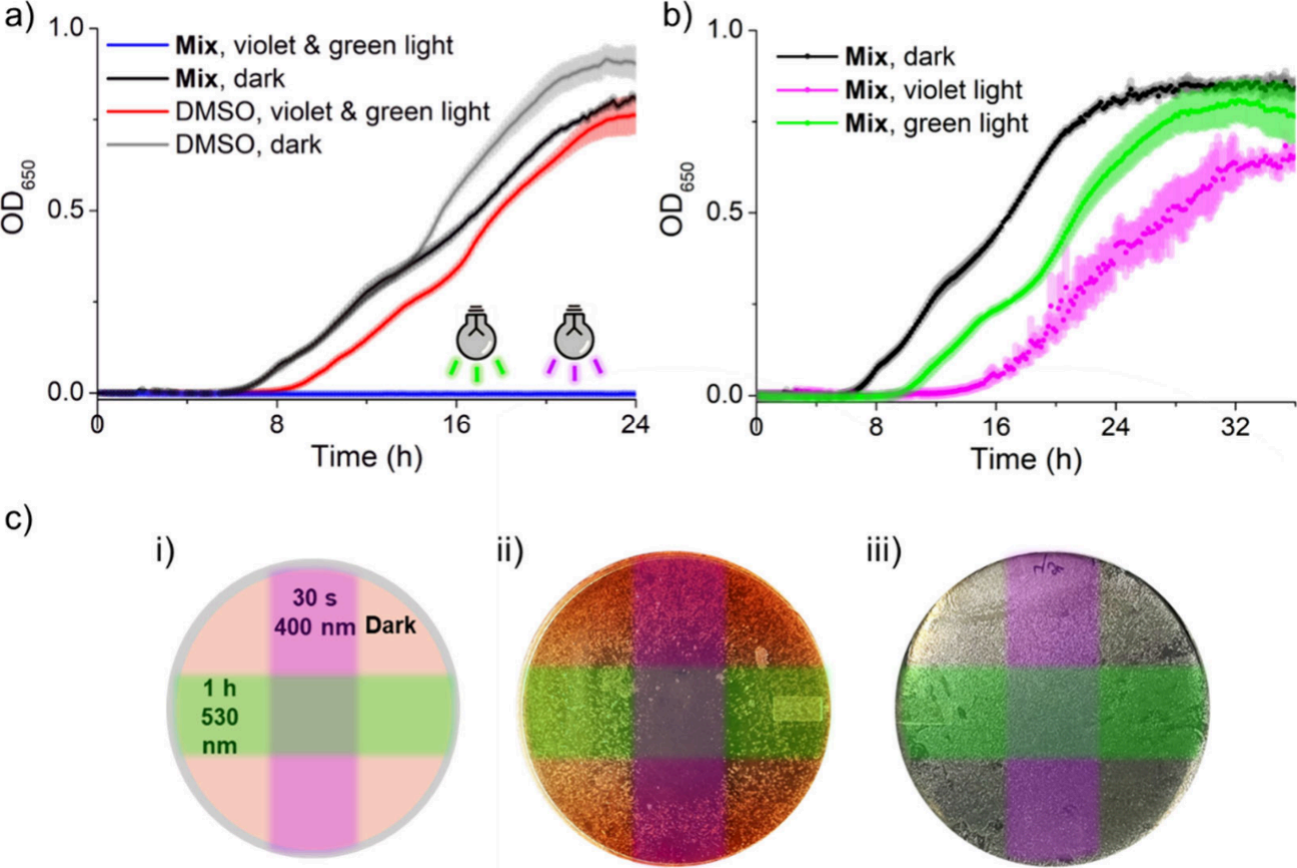
(a) The effect of irradiation on the growth of β-lactam resistant *E. coli* DH5α. Irradiation with light of both colors resulted in bacterial growth inhibition. (b) Irradiation with solely one of the two colors allowed for bacterial growth. (c) Spatial control of bacterial growth in a dual-color system. (i) Schematic representation of the layout of the plate experiment; two orthogonal slits allowed for an overlap of the two irradiation colors solely in the middle of the plate. Note that green light illumination took place from the top of the Petri dish (as in Figure 4), whereas violet light illumination was performed from the bottom of the dish. (ii) Results of the plate experiment with **Penicillin-PPG** and **Tazobactam-PPG** (80 and 180 μM, respectively). (iii) Control for light cytotoxicity (containing 80 μM penicillin).

Next, we aimed to study whether spatial resolution could be achieved through the dual-color dependent activation in a Petri-dish experiment. Using aluminum stickers, the lid and bottom of a Petri-dish containing inoculated LB-agar with **Penicillin-PPG** and **Tazobactam-PPG** (80 and 180, μM respectively) were covered such that slits were created allowing light to pass through in the middle. The plate was placed in a clamp and irradiated from above through the slit on the lid with green light (λ = 530 nm) for 1 h and through the slit on the bottom with violet light (λ = 400 nm) for 30 s (Figure 9c­(i), SI Section 3.3). After incubation at 37 °C overnight, the plate showed inhibition of bacterial growth solely in the middle of the plate where the illumination with green and violet light overlapped (Figure 9c­(ii)). In the areas that were kept in the dark or only irradiated with one color of light, bacterial growth occurred, albeit some inhibition of bacterial growth was observed slightly beyond the irradiated cross-section due to diffusion of uncaged compounds and/or incomplete shielding by the aluminum covers. These results indicate that the desired dual-color dependent activation of the antimicrobial effect was achieved. Irradiation of a control plate lacking both **Penicillin-PPG** and **Tazobactam-PPG**, but supplemented with penicillin (180 μM), did not result in inhibition of bacterial growth (Figure 9c­(iii)). Therefore, we conclude that the observed growth inhibition stems from the irradiation-dependent release of penicillin and tazobactam from both **Penicillin-PPG** and **Tazobactam-PPG**.

These results show that bacterial growth inhibition, depending on two colors of light, can be achieved spatially on a Petri dish. Therefore, in the future, if an effective three-dimensional model for bacterial growth can be constructed, the use of **Penicillin-PPG** and **Tazobactam-PPG** might allow for three-dimensional control over bacterial growth.

## Discussion and Outlook

This study presents the design of the novel photoactivatable antibiotic **Penicillin-PPG** and showcases its use to spatially control bacterial growth, achieve light-dependent inhibition of biofilm formation, and treat bacterial infection *in vivo*. Importantly, **Penicillin-PPG** displays antimicrobial activity only upon green light activation, which implies that this novel antimicrobial has a low propensity to elicit AMR. Photocaged antibiotics may thus become a valuable addition to the currently available arsenal of clinically applied antibiotics.

Both the liquid culture assays and the agar plate assays showed nearly 100% growth inhibition of *E. coli* by **Penicillin-PPG** activated with green light, whereas growth of *E. coli* was not inhibited by **Penicillin-PPG** in the dark. This highlights the reliability of **Penicillin-PPG** to remain completely inactive prior to light-driven activation. Furthermore, our investigations showed that activated **Penicillin-PPG** can kill 97% of the *S. epidermidis* bacteria in a biofilm. The fact that not all *S. epidermidis* bacteria in the biofilm were eliminated could relate to the experimental setup, where the plate containing all coverslips carrying the biofilms was irradiated by only one LED irradiation source, resulting in a suboptimal activation of **Penicillin-PPG** across the entire biofilm surface. However, biofilms of *S. epidermidis* are known for their high resistance to antibiotics, possibly because the biofilm-resident bacteria are not growing, whereas penicillin will kill only growing bacteria.^45^ Lastly, the treatment of *S. aureus*-infected *G. mellonella* larvae with green light-activated **Penicillin-PPG** shows that the photocaged penicillin can also be applied successfully in a living system. The results imply that the light-activated **Penicillin-PPG** reduced the burden of living bacteria *in vivo* either by direct killing of the growing bacteria or by reducing them to such an extent that they could be cleared by the larval immune defenses.^36^

Furthermore, another bioactive compound, tazobactam, was photocaged with a water-soluble coumarin PPG (**Tazobactam-PPG**). The absorption spectra of **Tazobactam-PPG** and **Penicillin-PPG** displayed a high degree of orthogonality and their uncaging could be performed with high selectivity depending on the wavelength of light used for irradiation. A sample containing **Penicillin-PPG** and **Tazobactam-PPG** required irradiation with both blue and green light to inhibit growth of a resistant *E. coli* strain in LB. This effect was also demonstrated spatially in two dimensions on an agar plate where only the overlap of the blue and green beams of light resulted in bacterial growth inhibition, illustrating that these compounds can potentially be used in the future for controlling antimicrobial activity in three dimensions. Here one has to bear in mind that the required irradiation times will depend on the required compound concentrations and the efficiency of the respective photocages. Ideally, efficient cages could be used that undergo chromophore destruction after irradiation, thereby increasing light penetration through the sample and reducing irradiation times.^31,46,47^

Whether photocaged antibiotics, such as **Penicillin-PPG**, can be applied effectively in the human body will largely depend on whether sites of infection can be sufficiently illuminated with green light. Here one needs to consider the fact that even infrared light, which has the highest tissue penetration, will only pass through about 1 cm of human tissue.^48^ Insufficient light penetration will therefore become a major hurdle when deep-seated infections need to be treated with photocaged antibiotics. In this context, placement of a CT-guided light source, for example, an endoscope, in the vicinity of the infected body site might be a solution. However, this will put patients at risk for the possible introduction of new infections. The feasibility of such an approach was recently demonstrated in a post-mortem setup, where a *S. epidermidis* biofilm on an infected knee prosthesis was imaged with a fluorescent conjugate of vancomycin and the near-infrared dye IRDye800CW using an arthroscope.^49^ To elicit fluorescence of the conjugate, it needed to be illuminated with a light beam that was guided through the arthroscope. In the case of a photocaged antibiotic, a readout for the determination of the amount of antibiotic that has been released at the site of infection would be desirable in order to assess whether the correct concentration of the antibiotic above the MIC of the targeted bacteria has been reached. A potential strategy through which this could be achieved is the use of another type of PPG that becomes fluorescent upon payload release.^42,50^ Then, based on the detected fluorescence, the degree of uncaging and, therefore, the amount of released antibiotic can be approximated. Furthermore, the combined use of photoactivatable antibiotics with aPDT, which relies on the light-induced generation of reactive oxygen species that kill bacteria, could be explored. As recent investigations have shown that a PDT is highly effective in destroying the outer layers of biofilms,^51^ this setup could be interesting in the context of implant-related infections. Altogether, the results of this study highlight the great potential of photopharmacology for light-dependent killing and the inhibition of bacterial growth *in vitro* and *in vivo*.

## Supplementary Material



## Notes

Synthetic procedures, NMR spectra, photochemical data, and detailed procedures for the biological experiments (PDF)
