# Supplementary material for: Green-Light-Activatable Penicillin for Light-Dependent Spatial Control of Bacterial Growth, Biofilm Formation, and In Vivo Infection Treatment
Source: ACS Cent Sci. 2025 Jun 11;11(7):1083–93. doi: 10.1021/acscentsci.5c00437 (PMC12291127; doi:10.1021/acscentsci.5c00437)
Supplement: Supplementary file 1 [file oc5c00437_si_001.pdf]

Supporting Information

Green-light-activatable penicillin for light-dependent spatial control of bacterial growth, biofilm formation and in vivo infection treatment

Albert Marten Schulte,[1,2] Jorrit W. A. Schoenmakers,[3] Marleen van Oosten,[3] Paul C. Jutte,[4] Jan Maarten van Dijl,[3*] Wiktor Szymanski [1,2,5,*] and Ben L. Feringa[1,2,*]

[1] Centre for Systems Chemistry, Stratingh Institute for Chemistry, Faculty for Science and Engineering, University of Groningen, Nijenborgh 4, 9747 AG Groningen, The Netherlands;
[2] Department of Medicinal Chemistry, Photopharmacology and Imaging, Groningen Research Institute of Pharmacy, University of Groningen, Antonius Deusinglaan 1, 9713 AV, Groningen, The Netherlands;
[3] Department of Medical Microbiology and Infection Prevention, University of Groningen, University Medical Center Groningen, Hanzeplein 1, 9713 GZ Groningen; The Netherlands;
[4] Department of Orthopaedics, University of Groningen, University Medical Center Groningen, Hanzeplein 1, 9713 GZ Groningen, The Netherlands
[5] Department of Radiology, Medical Imaging Center, University Medical Center Groningen, University of Groningen, Hanzeplein 1, 9713 GZ Groningen; The Netherlands

Contents

General remarks 3
1. Synthetic procedures 4
1.1 Penicillin-PPG 4
1.2 Tazobactam-PPG 6
1.3 NMR-spectra 9
2. Photochemistry 19
2.1 General 19
2.2 ^{1}H-NMR spectra of uncaging of Penicillin-PPG and Tazobactam-PPG 19
2.3 UV-Vis spectra for the uncaging process of Tazobactam-PPG 20
2.4 UPLC-MS traces of uncaging Tazobactam-PPG 21
2.5 Molar absorptivity coefficients of Penicillin-PPG and Tazobactam-PPG 22
2.6 UPLC-MS traces of uncaging of Penicillin-PPG and Tazobactam-PPG 23
3. Biological experiments 25
3.1 MIC-value determination 25

3.1.1 Benzylpenicillin towards *E. coli* DH5α 25
3.1.2 Benzylpenicillin and tazobactam towards *E. coli* DH5α + PET vector 25
3.2 Light dependent inhibition of bacterial growth in liquid culture 26
3.2.1 Penicillin-PPG – *E. coli* DH5α 26
3.2.2 Penicillin-PPG and Tazobactam-PPG – *E. coli* DH5α + PET-vector 26
3.3 Petri-dish experiments 27
3.3.1 Penicillin-PPG – *E. coli* DH5α 27
3.3.2 Penicillin-PPG and Tazobactam-PPG – *E. coli* DH5α + PET-vector 27
3.4 Prevention of biofilm formation using Penicillin-PPG 27
3.5 In vivo treatment of bacterial infection with green light-activated Penicillin-PPG 29
4. References 30

General remarks

All reactions were performed without excluding moisture or air, unless stated otherwise. Standard Schlenk techniques were used for reactions requiring an inert atmosphere (using nitrogen as the inert gas). Reagents were purchased from commercial suppliers (Sigma-Aldrich, TCI etc.) and used without further purification. Solvents were purchased from Boom B.V. or Sigma-Aldrich. Flash chromatography was performed on silica gel (Supelco, silica gel 60) with a particle size of 40-64 µm. Thin-layer chromatography (TLC) analysis was conducted on TLC plates with a silica gel matrix (Supelco, silica gel 60) with detection by UV-light (254 or 366 nm).

Nuclear magnetic resonance (NMR) spectra were recorded on an Agilent Technologies 400-MR (400/54 Premium Shielded) spectrometer (400 MHz for ^{1}H nucleus, 101 MHz for ^{13}C nucleus). Deuterated solvents (DMSO-d_6 and $CDCl_3$) were purchased from Sigma-Aldrich. The chemical shift of compound resonances is given in parts per million (ppm, δ) and reported relative to the residual solvent proton or carbon resonance (DMSO-d_6 and $CDCl_3$). All spectra were measured at ambient temperature. ^{1}H-NMR data are reported as: chemical shift, multiplicity (s = singlet, d = doublet, t = triplet, q = quartet, m = multiplet, dd = doublet of doublets, dt = doublet of triplets, dq = doublet of quartets, br = broad), coupling constants (J) given in Hz, and integration. ^{13}C-NMR spectra were conducted with proton decoupling and the chemical shifts are reported.

High resolution mass spectra (HRMS) were recorded on a Thermofisher LTQ Orbitrap XL with eluent MeOH (0.1 % TFA) and flow rate of 0.15 mL min^{-1} in positive (ACPI/ESI) mode. UV-vis spectra were recorded with an Agilent 8543 spectrophotometer. Raw data were processed using Agilent UV-vis Chemstation B.02.01 SP1, Spectragryph 1.2, OriginPro 8.5 and MS Excel.

1. Synthetic procedures

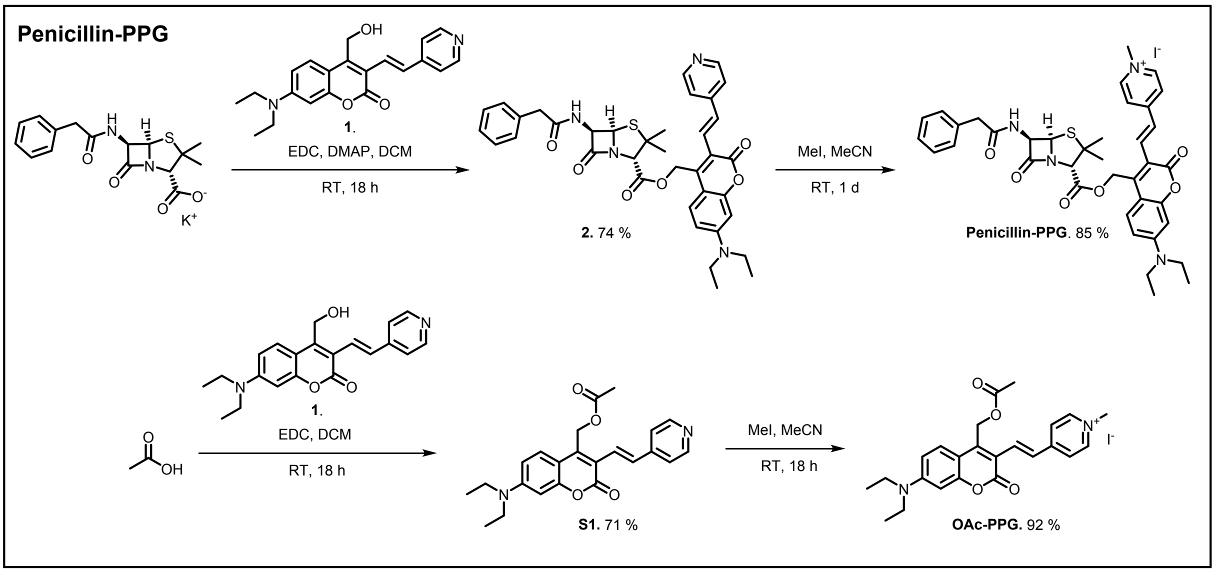

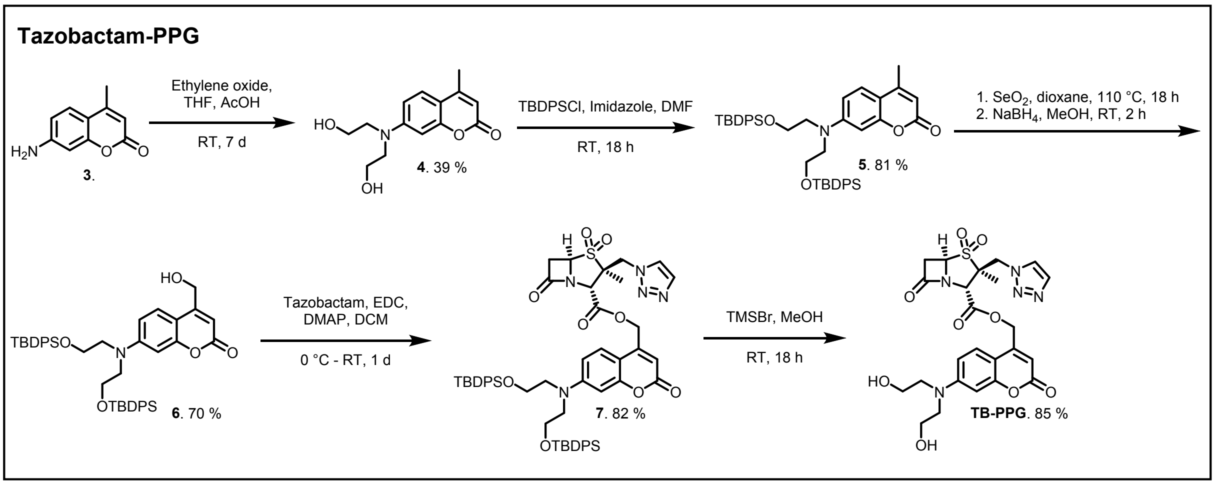

1.1 Penicillin-PPG

Compound 1 was synthesized according to a literature procedure. [1]

Compound 2 (7-(diethylamino)-2-oxo-3-((E)-2-(pyridin-4-yl)vinyl)-2H-chromen-4-yl)methyl (2S,5R,6R)-3,3-dimethyl-7-oxo-6-(2-phenylacetamido)-4-thia-1-azabicyclo[3.2.0]heptane-2-carboxylate):

Benzylpenicillin potassium salt (478 mg, 1.28 mmol, 5.00 eq.), compound **1** (90 mg, 0.26 mmol, 1.00 eq.) and EDC (246 mg, 1.28 mmol, 5.00 eq.) were dissolved in dry DCM (3.2 mL) under nitrogen atmosphere. DMAP (12 mg, 0.10 mmol, 0.40 eq.) was added and the mixture was stirred at room temperature in the dark for 18 h. The mixture was diluted with DCM and washed with saturated aqueous $NaHCO_3$ and water. After both washing steps, the aqueous layer was back-extracted with DCM, and the organic layers were combined and taken to the next step. The combined organic layers were washed with brine, dried over $MgSO_4$, and concentrated under reduced pressure. Silica gel chromatography (DCM/Acetone 9:1 to 8:2) yielded the pure product as a red powder (126 mg, 74 %). **^{1}H-NMR** (400 MHz, $CDCl_3$) δ 8.57 (d, *J* = 5.4 Hz, 2H), 7.79 (d, *J* = 15.9 Hz, 1H), 7.56 – 7.47 (m, 4H), 7.39

– 7.28 (m, 3H), 7.25 (d, *J* = 6.7 Hz, 2H), 6.66 (dd, *J* = 9.2, 2.6 Hz, 1H), 6.52 (d, *J* = 2.6 Hz, 1H), 6.03 (d, *J* = 9.0 Hz, 1H), 5.67 – 5.55 (m, 2H), 5.48 – 5.38 (m, 2H), 4.41 (s, 1H), 3.62 (s, 2H), 3.46 (q, *J* = 7.2 Hz, 4H), 1.35 (s, 3H), 1.34 (s, 3H), 1.26 – 1.22 (m, 6H). **^{13}C-NMR** (101 MHz, $CDCl_3$) δ 173.6, 170.4, 167.3, 160.4, 155.5, 151.2, 149.5, 146.0, 142.8, 133.9, 132.1, 129.7, 129.3, 127.8, 126.3, 125.1, 121.3, 116.8, 109.7, 107.9, 97.6, 70.4, 68.3, 64.6, 59.0, 45.1, 43.5, 32.0, 31.1, 26.9, 12.6. **HRMS** (ESI): calc. for $C_{37}H_{39}N_4O_6S^+$ (M+H$^+$): 667.2585; found: 667.2580.

Penicillin-PPG (4-((*E*)-2-(7-(diethylamino)-4-((((2S,5R,6R)-3,3-dimethyl-7-oxo-6-(2-phenylacetamido)-4-thia-1-azabicyclo[3.2.0]heptane-2-carbonyl)oxy)methyl)-2-oxo-2H-chromen-3-yl)vinyl)-1-methylpyridin-1-ium iodide):

Procedure adapted from Bojtár *et al.*[1]

To a solution of compound **2** (66 mg, 0.10 mmol, 1.0 eq.) in MeCN (1.1 mL) was added MeI (31 µL, 0.50 mmol, 5.00 eq.). The solution was stirred at room temperature in the dark for 1 d, diluted with Et_2O (10 mL) and filtered. The residue that remained in the flask was dissolved in a minimal amount of DCM, diluted with Et_2O (5 mL) and filtered. The residue on the filter was washed with Et_2O and dried under vacuum to yield Penicillin-PPG as a dark red solid (68 mg, 85 %). **^{1}H-NMR** (400 MHz, D_2O/DMSO-d_6 1:1) δ 8.38 (d, *J* = 6.5 Hz, 2H), 7.88 (d, *J* = 6.5 Hz, 2H), 7.78 – 7.59 (m, 3H), 7.28 – 7.03 (m, 5H), 6.73 (d, *J* = 9.4 Hz, 1H), 6.45 (s, 1H), 5.58 (s, 2H), 5.24 (d, *J* = 4.0 Hz, 1H), 5.15 (d, *J* = 4.1 Hz, 1H), 4.38 (s, 1H), 4.02 (s, 3H), 3.57 – 3.15 (m, 6H), 1.32 (s, 3H), 1.13 – 0.95 (m, 9H). **^{13}C-NMR** (101 MHz, $CDCl_3$) δ 173.2, 170.7, 167.3, 160.0, 156.2, 154.4, 152.2, 147.6, 144.7, 134.2, 134.1, 129.7, 129.1, 127.6, 127.5, 126.3, 124.3, 114.0, 110.3, 108.1, 97.3, 70.3, 68.2, 64.7, 59.1, 59.0, 48.3, 45.2, 43.3, 32.2, 26.9, 12.7. **HRMS** (ESI): calc. for $C_{38}H_{41}N_4O_6S^+$ (M$^+$): 681.2741; found: 681.2738.

Compound S1 (*E*)-(7-(diethylamino)-2-oxo-3-(2-(pyridin-4-yl)vinyl)-2H-chromen-4-yl)methyl acetate:

Compound **1** (65 mg, 0.19 mmol, 1.00 eq.) and EDC (178 mg, 0.93 mmol, 5.00 eq.) were dissolved in dry DCM (2.3 mL) under nitrogen atmosphere. AcOH (53 µL, 0.93 mmol, 5.00 eq.) was added and the mixture was stirred at room temperature in the dark for 18 h. The mixture was diluted with DCM and washed with saturated aqueous $NaHCO_3$ and water. After both washing steps, the aqueous layer was back-extracted with DCM, and the combined organic layers were taken to the next step. The combined organic layers were washed with brine, dried over $MgSO_4$, and concentrated under reduced pressure. Silica gel chromatography (DCM/MeOH 98:2 to 97:3) yielded the pure product as a red powder (52 mg, 71 %). **^{1}H-NMR** (400 MHz, $CDCl_3$) δ 8.57 (d, *J* = 5.0 Hz, 2H), 7.73 (d, *J* = 16.0 Hz, 1H), 7.59 – 7.49 (m, 2H), 7.45 (d, *J* = 5.5 Hz, 2H), 6.65 (dd, *J* = 9.2, 2.6 Hz, 1H), 6.52 (d, *J* = 2.6 Hz, 1H), 5.44 (s, 2H), 3.44 (q, *J* = 7.1 Hz, 4H), 2.13 (s, 3H), 1.23 (t, *J* = 7.1 Hz, 6H). **^{13}C-NMR** (101 MHz, $CDCl_3$) δ 170.6, 160.7, 155.5, 151.0, 149.5, 146.2, 144.3, 131.8, 126.6, 125.5, 121.3, 116.7, 109.6, 108.3, 97.5, 58.0, 45.0, 21.0, 12.7. **HRMS** (ESI): calc. for $C_{23}H_{25}N_2O_4^+$ (M+H$^+$): 383.1809; found: 393.1810.

OAc-PPG (*E*)-4-(2-(4-(acetoxymethyl)-7-(diethylamino)-2-oxo-2H-chromen-3-yl)vinyl)-1-methylpyridin-1-ium:

Procedure adapted from Bojtár *et al.*[1]

To a solution of compound **S1** (35 mg, 0.09 mmol, 1.00 eq.) in MeCN (1 mL) was added MeI (28 µL, 0.45 mmol, 5.00 eq.). The solution was stirred at room temperature in the dark for 18 h, diluted with Et_2O (10 mL) and filtered. The residue that remained in the flask was dissolved in a minimal amount of DCM, diluted with Et_2O (5 mL) and filtered. The residue on the filter was washed with Et_2O and dried under vacuum to yield **OAc-PPG** as a dark red solid (44 mg, 92 %). **^{1}H-NMR** (400 MHz, $CDCl_3$) δ 8.99 (d, *J* = 6.1 Hz, 2H), 7.99 (s, 4H), 7.69 (d, *J* = 9.3 Hz, 1H), 6.68 (dd, *J* = 9.3, 2.6 Hz, 1H), 6.46 (d, *J* = 2.5 Hz, 1H), 5.53 (s, 2H), 4.57 (s, 3H), 3.46 (q, *J* = 7.1 Hz, 4H), 2.11 (s, 3H), 1.24 (t, *J* = 7.1 Hz, 6H). **^{13}C-NMR** (101 MHz, $CDCl_3$) δ 170.6, 160.1, 156.3, 154.8, 152.2, 148.7, 144.8, 134.2, 127.8, 126.2, 124.1, 114.0, 110.3, 108.5, 97.3, 57.5, 48.5, 45.2, 21.2, 12.7. **HRMS** (ESI): calc. for $C_{24}H_{27}N_2O_4^+$ (M^+): 407.1965; found: 407.1966.

1.2 Tazobactam-PPG

Compound 4 (7-(bis(2-hydroxyethyl)amino)-4-methyl-2H-chromen-2-one):

Procedure adapted from Huang *et al.*[2]

Compound **3** (2.80 g, 15.98 mmol, 1.00 eq.) was suspended in THF (28 mL) and AcOH (94 mL) was added. The mixture was cooled to 0 °C, and a solution of ethylene oxide in THF (2.6 M, 24.6 mL, 63.9 mmol, 4.00 eq.) was added. The mixture was stirred at room temperature for 7 d and concentrated under reduced pressure to remove the volatiles. The residue was diluted with water (20 mL) and brine (40 mL) and extracted with EtOAc (4 x 100 mL). The combined organic layers were washed with brine, dried over $MgSO_4$, and evaporated under reduced pressure. The crude residue was purified by silica gel chromatography (EtOAc with 0.1% TEA to EA/MeOH 95:5 with 0.1 % TEA) to yield compound **4** (1.64 g, 39 %).**^{1}H-NMR** (400 MHz, CD_3OD) δ 7.49 (d, *J* = 9.0 Hz, 1H), 6.79 (dd, *J* = 9.1, 2.6 Hz, 1H), 6.58 (d, *J* = 2.6 Hz, 1H), 5.92 (s, 1H), 3.77 (t, *J* = 5.9 Hz, 4H), 3.63 (t, *J* = 6.0 Hz, 4H), 2.35 (s, 3H). **^{13}C-NMR** (101 MHz, CD_3OD) δ 164.7, 156.9, 156.2, 153.0, 127.0, 110.7, 110.7, 108.9, 98.8, 60.1, 54.8, 18.5. Spectra in accordance with literature.

Compound 5 (7-(bis(2-((tert-butyldiphenylsilyl)oxy)ethyl)amino)-4-methyl-2H-chromen-2-one):

To a solution of compound **4** (575 mg, 2.18 mmol, 1.00 eq.) in DMF (8 mL) was added TBDPSCl (2.10 mL, 8.08 mmol, 3.70 eq.) and imidazole (1.04 g, 15.29 mmol, 7.00 eq.). The mixture was stirred at RT for 12 h, poured into water and extracted with DCM (100 mL). The organic layer was separated and washed with brine, dried over $MgSO_4$, and concentrated under reduced pressure. The crude material was purified by silica gel chromatography (Pentane/EtOAc 9:1 to 8:2) to yield compound **5** as a yellow powder (1.31 g, 81 %). **^{1}H-NMR** (400 MHz, $CDCl_3$) δ 7.61 (dt, *J* = 6.8, 1.5 Hz, 8H), 7.46 – 7.37 (m, 4H), 7.37 – 7.29 (m, 8H), 7.18 (d, *J* = 8.9 Hz, 1H), 6.36 (d, *J* = 2.5 Hz, 1H), 6.32 (dd, *J* = 8.9, 2.6 Hz, 1H), 5.95

(d, *J* = 1.3 Hz, 1H), 3.78 (t, *J* = 6.1 Hz, 4H), 3.52 (t, *J* = 6.1 Hz, 4H), 2.32 (s, 3H), 1.04 (s, 18H). **^{13}C-NMR** (101 MHz, $CDCl_3$) δ 162.2, 155.9, 152.7, 151.0, 135.7, 133.3, 129.9, 127.9, 125.4, 109.6, 109.3, 108.8, 98.2, 60.8, 53.1, 26.9, 19.2, 18.5. **HRMS** (ESI): calc. for $C_{46}H_{54}NO_4Si_2$ ($M+H^+$): 740.3586; found: 740.3576.

Compound 6 7-(bis(2-((tert-butyldiphenylsilyl)oxy)ethyl)amino)-4-(hydroxymethyl)-2H-chromen-2-one:

To a solution of compound **5** (116 mg, 0.16 mmol, 1.00 eq.) in dioxane (4 mL) was added SeO_2 (35 mg, 0.31 mmol, 2.00 eq.). The mixture was stirred at 110 °C for 18 h, cooled, and filtered. The residue was washed with EtOAc. The filtrate was concentrated under reduced pressure and dissolved in MeOH (3 mL). $NaBH_4$ (12 mg, 0.31 mmol, 2.00 eq.) was added, the mixture was stirred at RT for 2 h and concentrated under reduced pressure. The residue was dissolved in DCM and the organic layer was washed with water, brine, dried over $MgSO_4$ and concentrated under reduced pressure. The crude material was purified by silica gel chromatography (Pentane/EtOAc 8:2 to 7:3) to yield compound **6** as an orange powder (83 mg, 70 %). **^{1}H-NMR** (400 MHz, $CDCl_3$) δ 7.63 – 7.53 (m, 8H), 7.44 – 7.36 (m, 4H), 7.36 – 7.27 (m, 8H), 7.11 (d, *J* = 9.0 Hz, 1H), 6.36 (d, *J* = 2.6 Hz, 1H), 6.29 (dd, *J* = 9.0, 2.6 Hz, 1H), 6.26 (s, 1H), 4.80 (d, *J* = 1.4 Hz, 2H), 3.76 (t, *J* = 6.0 Hz, 4H), 3.51 (t, *J* = 6.0 Hz, 4H), 1.03 (s, 18H). **^{13}C-NMR** (101 MHz, $CDCl_3$) δ 162.4, 156.0, 154.3, 151.0, 135.7, 133.3, 130.0, 127.9, 124.2, 109.0, 106.9, 106.1, 98.5, 61.1, 60.8, 53.1, 26.9, 19.2. **HRMS** (ESI): calc. for $C_{46}H_{54}NO_5Si_2$ ($M+H^+$): 756.3535; found: 756.3531.

Compound 7 (7-(bis(2-((tert-butyldiphenylsilyl)oxy)ethyl)amino)-2-oxo-2H-chromen-4-yl)methyl (2*S*,3*S*,5*R*)-3-((1H-1,2,3-triazol-1-yl)methyl)-3-methyl-7-oxo-4-thia-1-azabicyclo[3.2.0]heptane-2-carboxylate 4,4-dioxide:

At 0 °C, under nitrogen atmosphere, compound **6** (350 mg, 0.46 mmol, 1.00 eq.), tazobactam (695 mg, 2.32 mmol, 5.00 eq.) and EDC (444 mg, 2.32 mmol, 5.00 eq.) were dissolved in dry DCM (10.6 mL) and DMAP (22 mg, 0.19 mmol, 0.40 eq.) was added. The mixture was stirred at RT for 1 d, diluted with DCM, and washed with sat. aq. $NaHCO_3$ (1 x) and water (1 x). The organic layer was dried over $MgSO_4$ and concentrated under reduced pressure. The crude material was purified by silica gel chromatography (DCM/acetone 97:3 to 93:7) to yield compound **7** as a yellow powder (396 mg, 82 %). **^{1}H-NMR** (400 MHz, $CDCl_3$) δ 7.73 (d, *J* = 1.1 Hz, 1H), 7.67 (d, *J* = 1.1 Hz, 1H), 7.63 – 7.53 (m, 8H), 7.45 – 7.37 (m, 4H), 7.33 (ddd, *J* = 8.2, 6.6, 4.1 Hz, 8H), 7.11 (d, *J* = 8.6 Hz, 1H), 6.40 (d, *J* = 8.5 Hz, 2H), 6.13 (s, 1H), 5.40 (d, *J* = 14.4 Hz, 1H), 5.23 (d, *J* = 14.3 Hz, 1H), 5.05 – 4.93 (m, 2H), 4.70 (s, 1H), 4.67 (dd, *J* = 4.3, 2.0 Hz, 1H), 3.75 (t, *J* = 5.9 Hz, 4H), 3.65 – 3.45 (m, 6H), 1.38 (s, 3H), 1.02 (s, 18H). **^{13}C-NMR** (101 MHz, $CDCl_3$) δ 169.9, 165.8, 161.3, 156.2, 151.6, 147.1, 135.6, 134.3, 133.2, 123.0, 127.9, 125.9, 124.2, 109.5, 108.1, 106.0, 98.6, 65.3, 63.8, 62.7, 60.8, 60.6, 53.1, 50.4, 39.3, 26.9, 19.2, 16.5. **HRMS** (ESI): calc. for $C_{56}H_{64}N_5O_9SSi_2$ ($M+H^+$): 1038.3958; found: 1038.3951.

Tazobactam-PPG

(7-(bis(2-hydroxyethyl)amino)-2-oxo-2H-chromen-4-yl)methyl (2*S*,3*S*,5*R*)-3-((1H-1,2,3-triazol-1-yl)methyl)-3-methyl-7-oxo-4-thia-1-azabicyclo[3.2.0]heptane-2-carboxylate 4,4-dioxide

Procedure adapted from Shah *et al.*[3]

Compound **7** (350 mg, 0.34 mmol, 1.00 eq.) was dissolved in MeOH (7 mL) and TMSBr (22 µL, 0.17 mmol, 0.50 eq.) was added. The mixture was stirred at RT for 18 h and concentrated under reduced pressure. The crude material was purified by silica gel chromatography (EA/MeOH 97:3 to 92:8) to yield **Tazobactam-PPG** as a yellow powder (161 mg, 85 %). **^{1}H-NMR** (400 MHz, CD_3OD) δ 8.01 (s, 1H), 7.75 (s, 1H), 7.54 (d, *J* = 9.0 Hz, 1H), 6.85 (dd, *J* = 9.1, 2.6 Hz, 1H), 6.67 (d, *J* = 2.5 Hz, 1H), 6.19 (s, 1H), 5.55 – 5.39 (m, 2H), 5.25 (d, *J* = 15.2 Hz, 1H), 5.09 – 4.96 (m, 3H), 3.77 (t, *J* = 5.8 Hz, 4H), 3.71 – 3.60 (m, 5H), 3.41 (d, *J* = 16.4 Hz, 1H), 1.40 (s, 3H). **^{13}C-NMR** (101 MHz, CD_3OD) δ 172.4, 167.6, 163.9, 157.4, 153.3, 150.9, 134.5, 128.2, 126.4, 111.1, 108.3, 107.7, 99.2, 66.2, 64.7, 63.8, 61.6, 60.1, 54.8, 51.6, 39.3, 16.7. **HRMS** (ESI): calc. for $C_{24}H_{27}N_5O_9S$ ($M+H^+$): 562.1602; found: 562.1585.

1.3 NMR-spectra

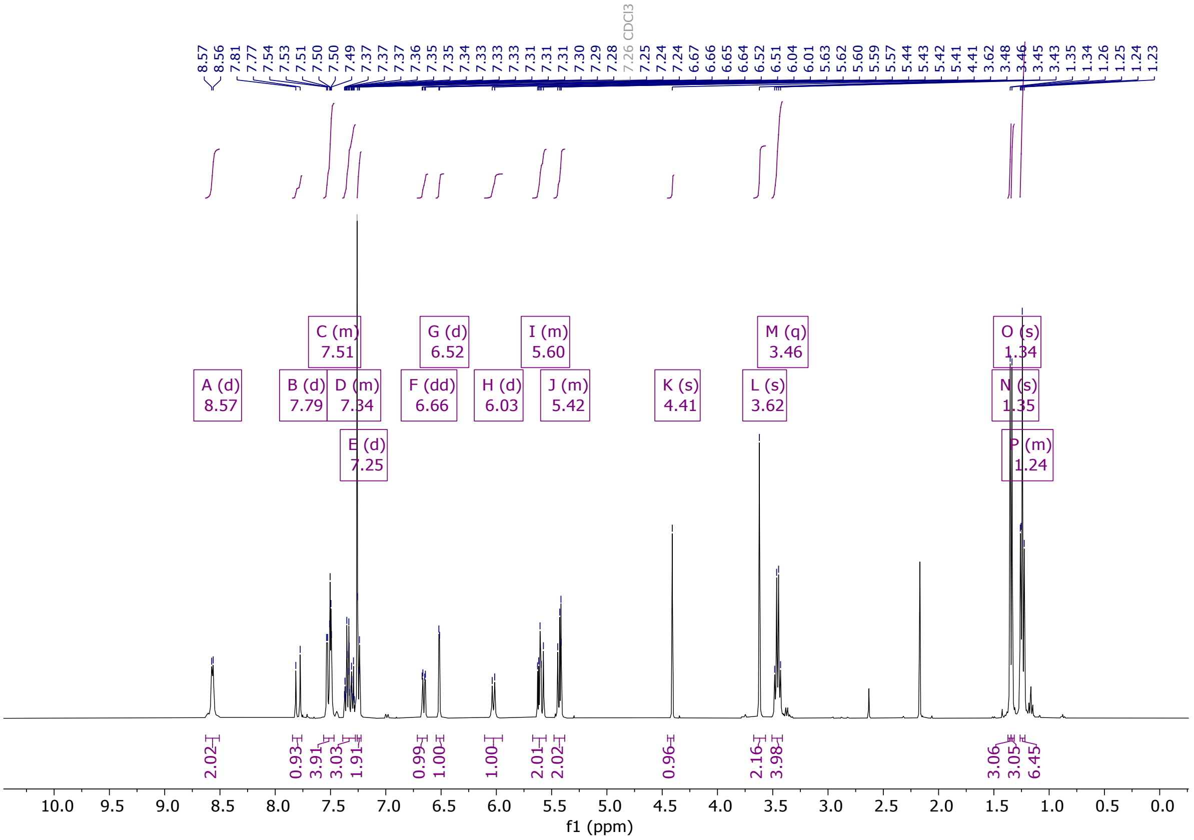

Figure S1. ^{1}H-NMR spectrum of compound **2** ($CDCl_3$).

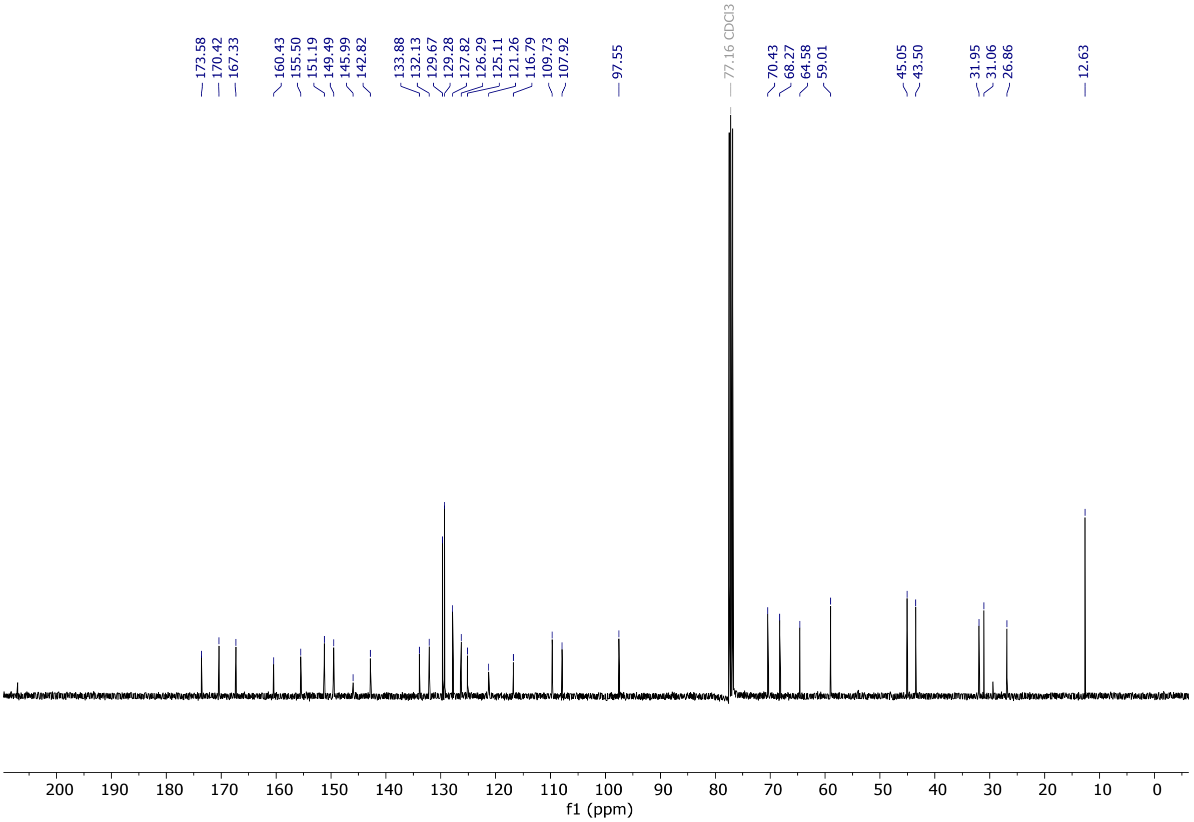

Figure S2. ^{13}C-NMR spectrum of compound **2** ($CDCl_3$)

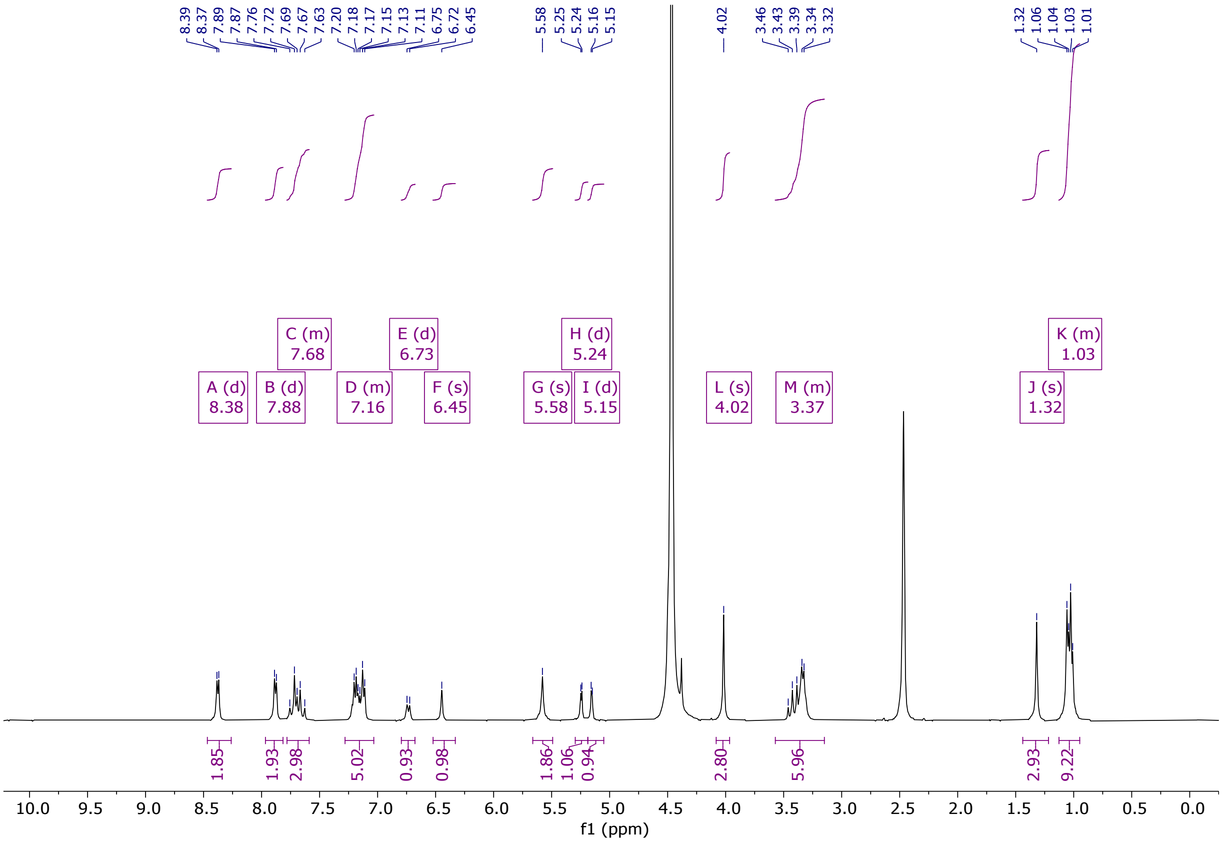

Figure S3. ^{1}H-NMR spectrum of **Penicillin-PPG** (DMSO-d_6/D_2O 1:1)

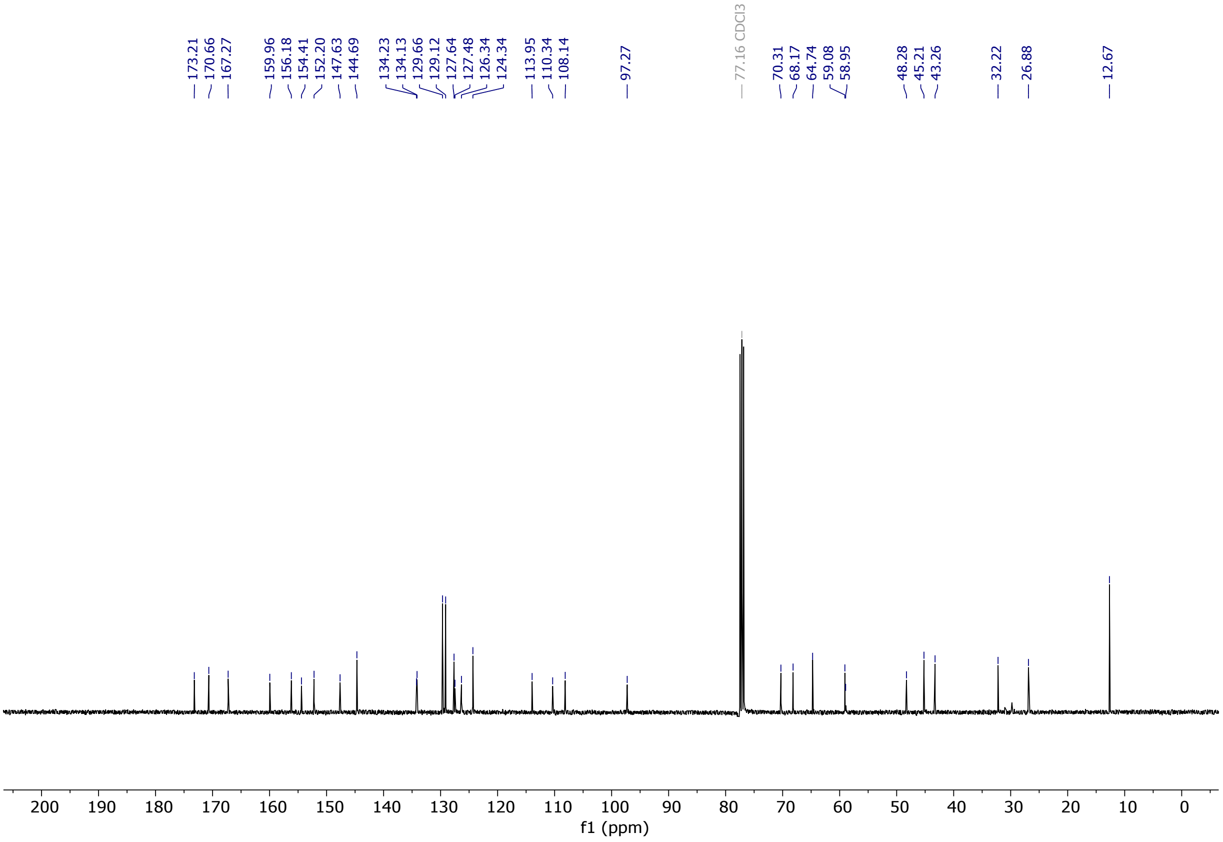

Figure S4. ^{13}C-NMR spectrum of **Penicillin-PPG** ($CDCl_3$)

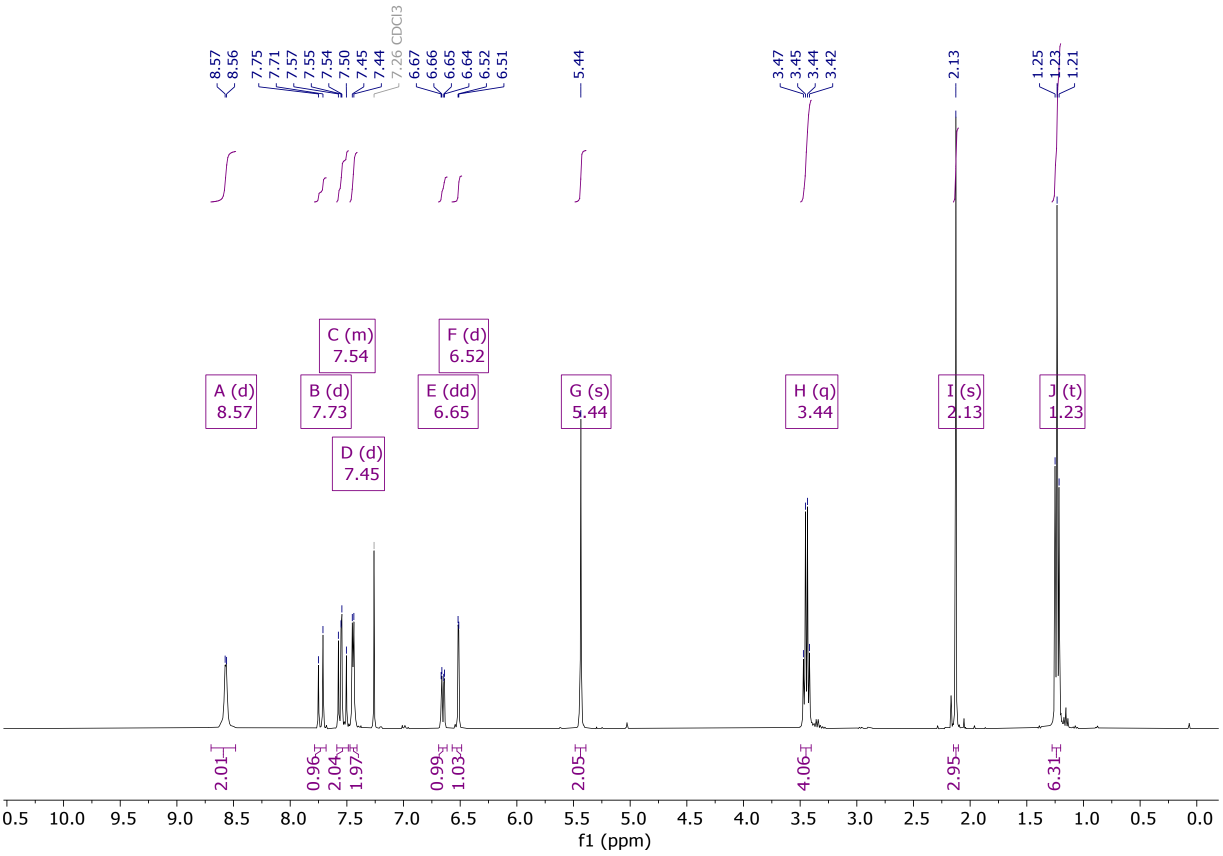

Figure S5. ^{1}H-NMR spectrum of compound **S1** ($CDCl_3$)

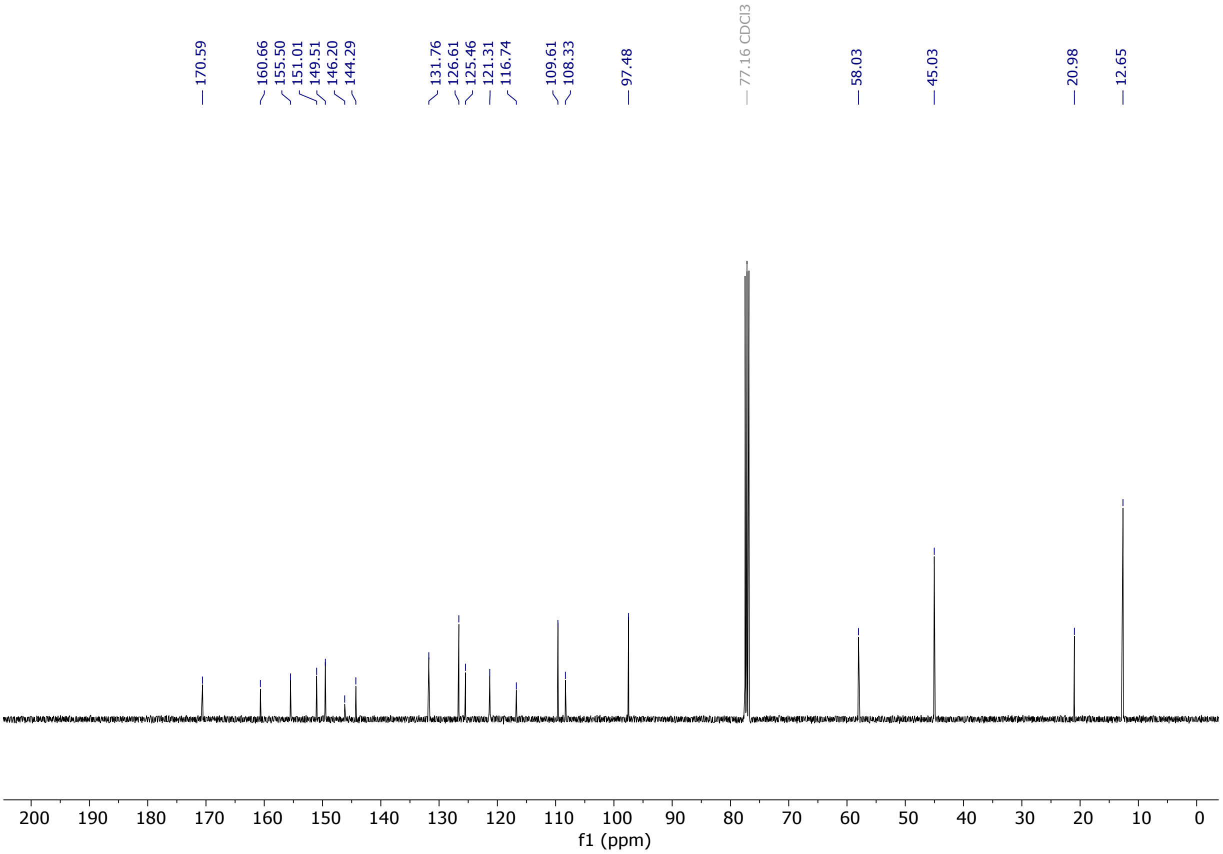

Figure S6. ^{13}C-NMR spectrum of compound **S1** ($CDCl_3$)

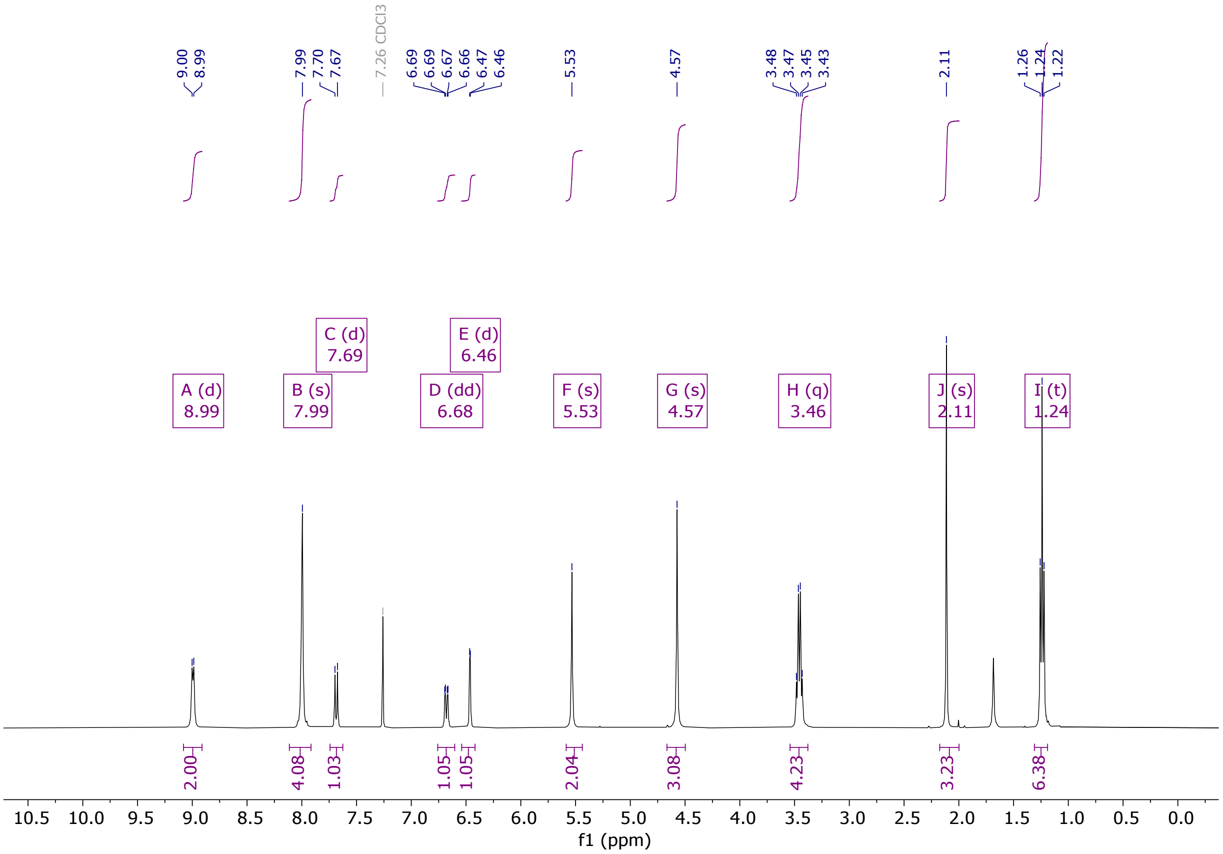

Figure S7. ^{1}H-NMR spectrum of **OAc-PPG** ($CDCl_3$)

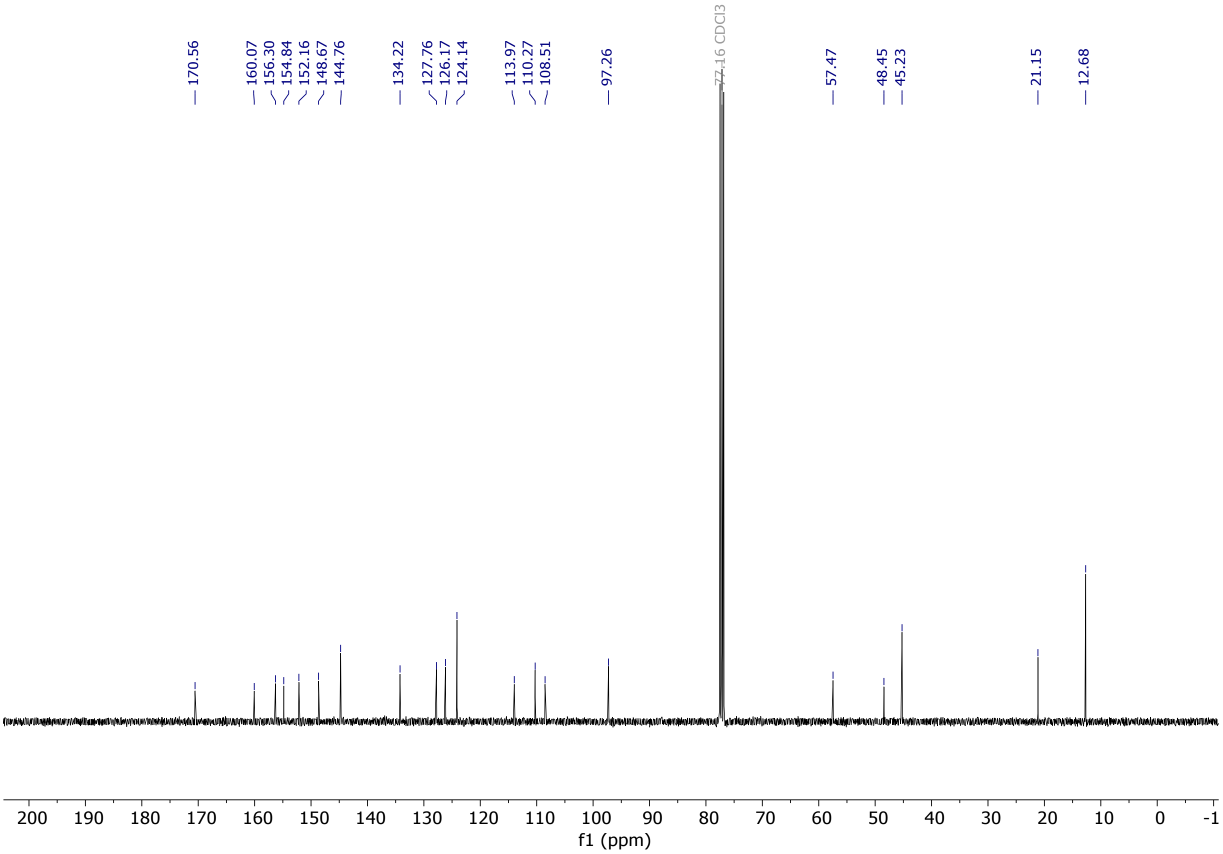

Figure S8. ^{13}C-NMR spectrum of **OAc-PPG** ($CDCl_3$)

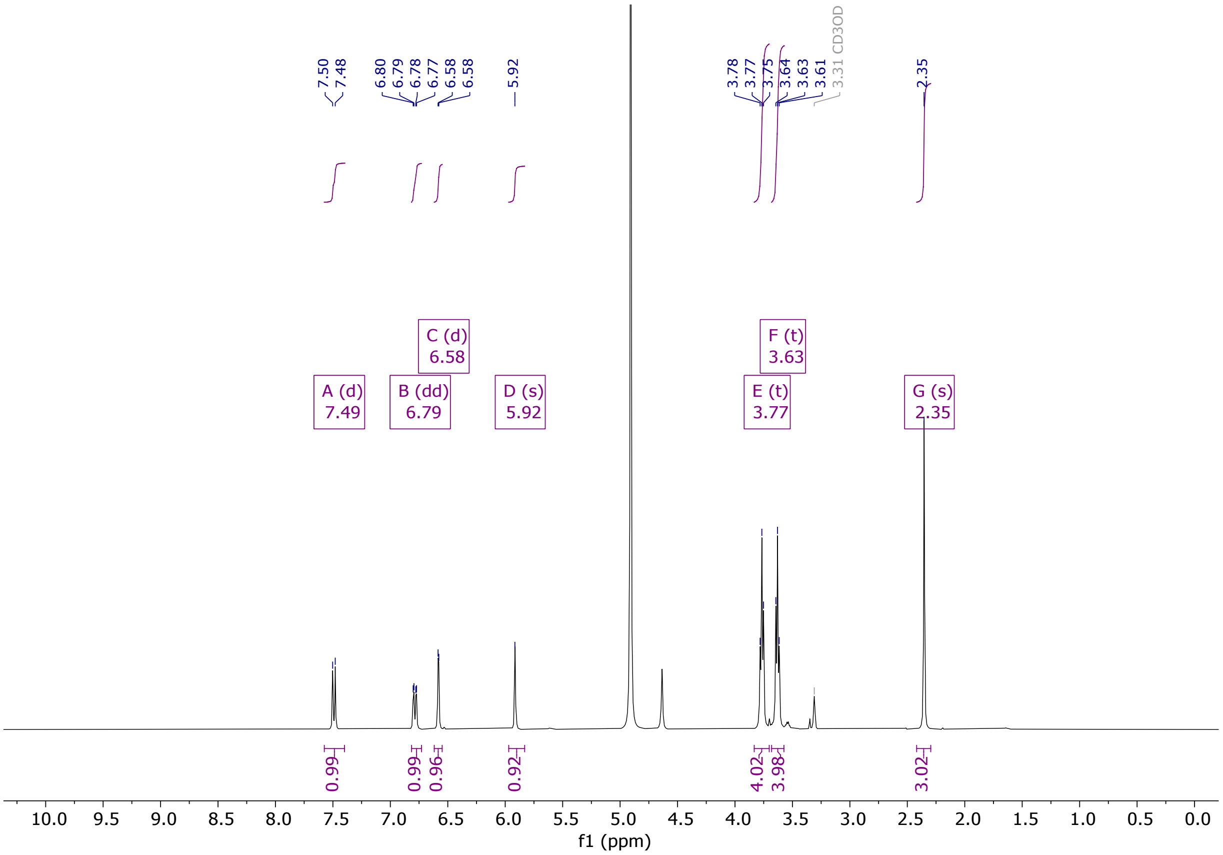

Figure S9. ^{1}H-NMR spectrum of compound **4** (CD_3OD)

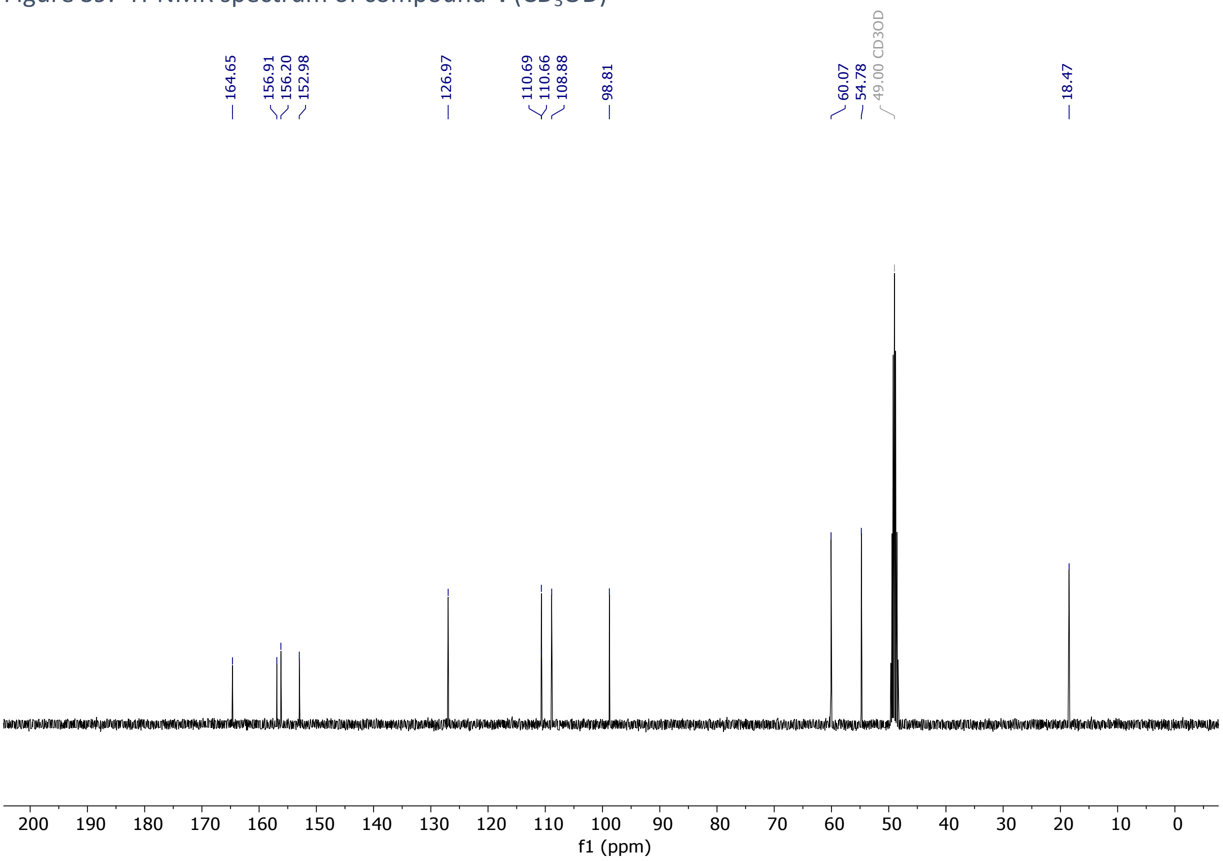

Figure S10. ^{13}C-NMR spectrum of compound **4** (CD_3OD)

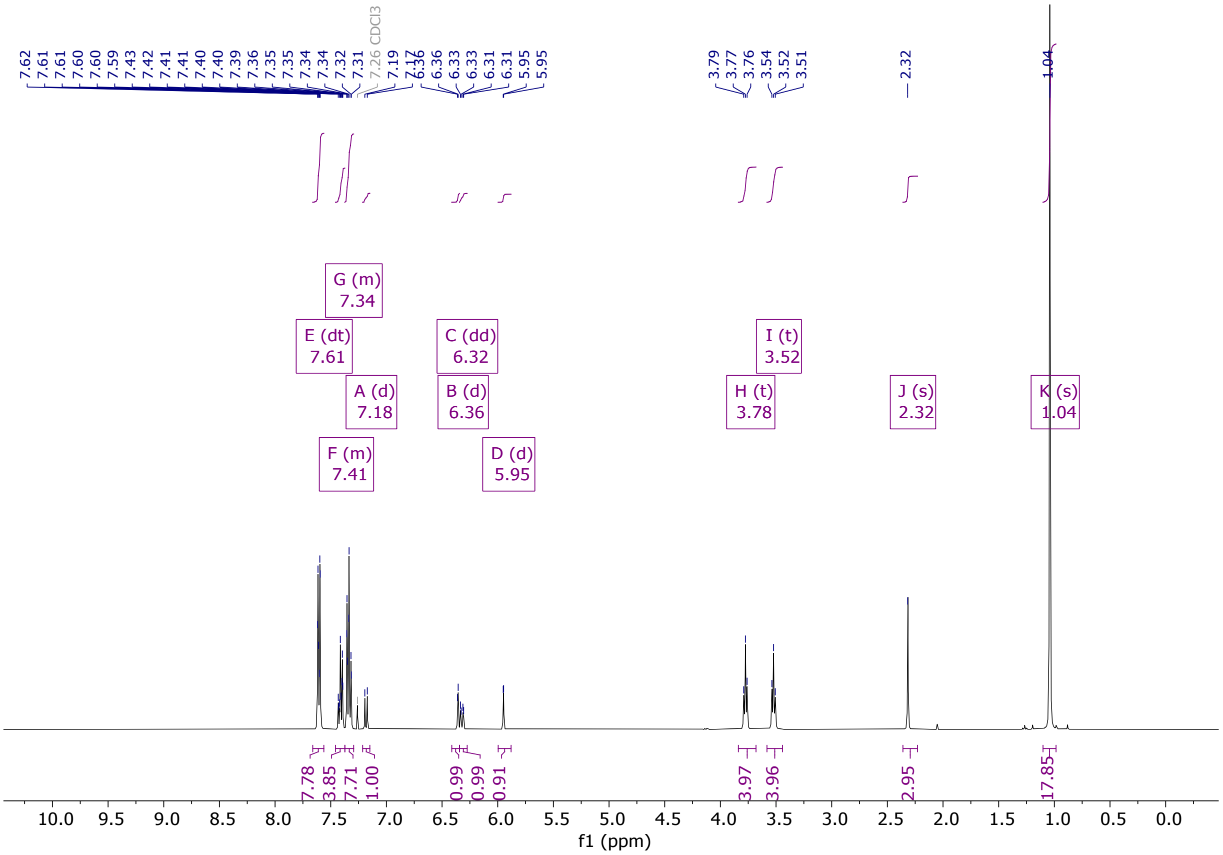

Figure S11. ^{1}H-NMR spectrum of compound **5** ($CDCl_3$)

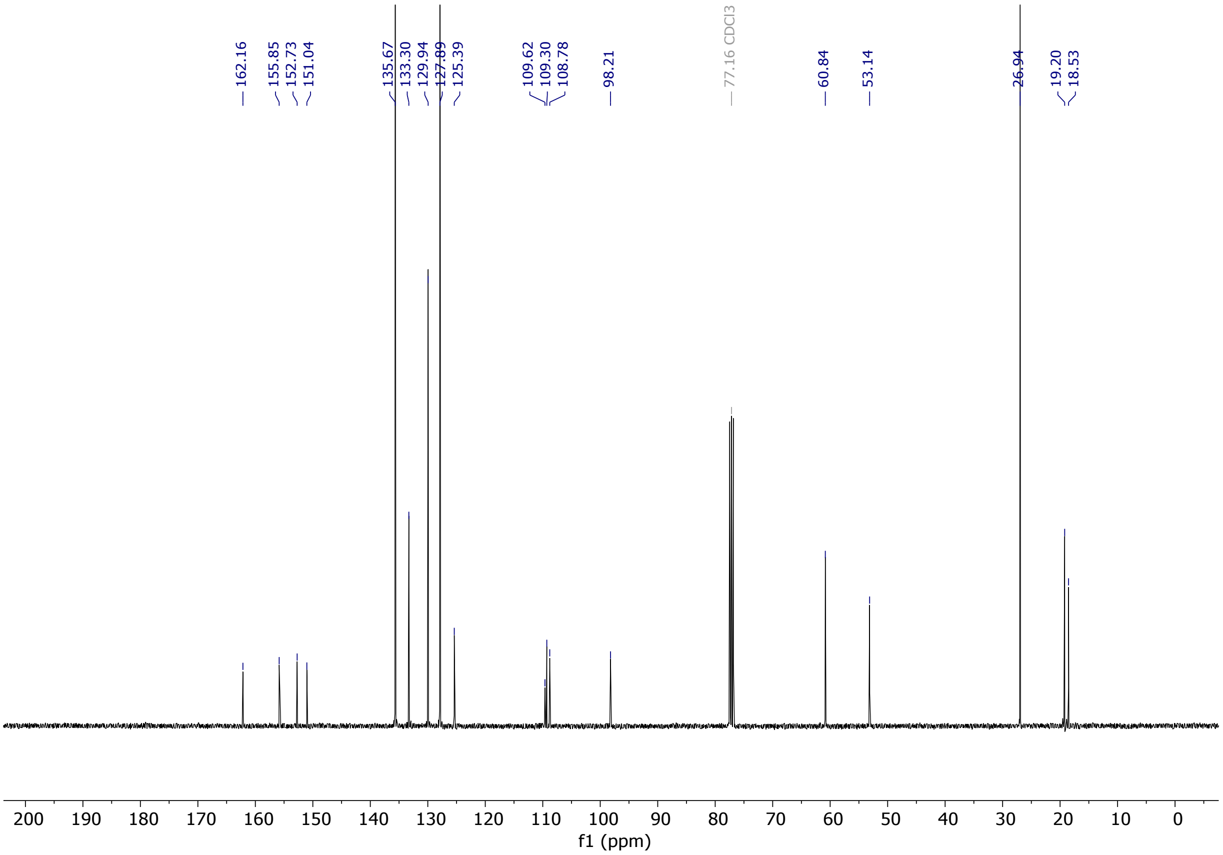

Figure S12. ^{13}C-NMR spectrum of compound **5** ($CDCl_3$)

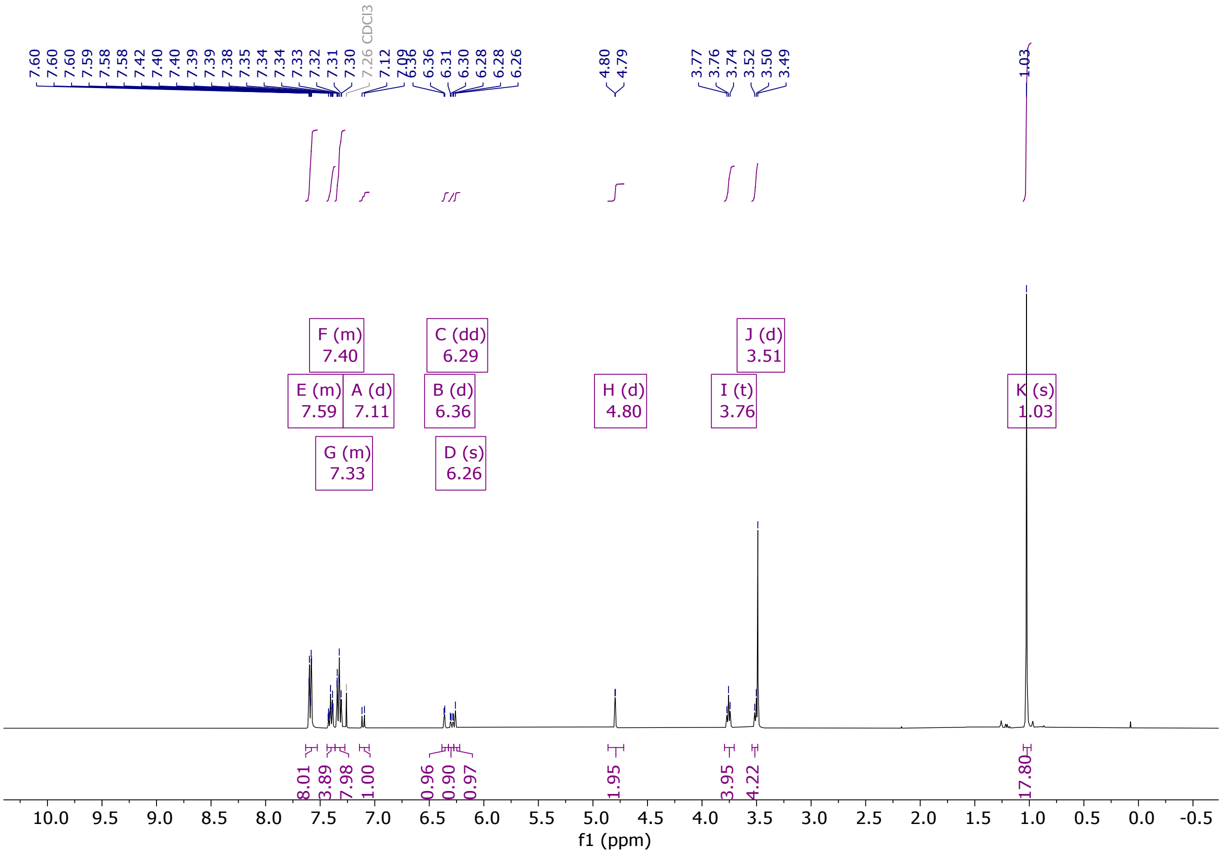

Figure S13. ^{1}H-NMR spectrum of compound **6** ($CDCl_3$)

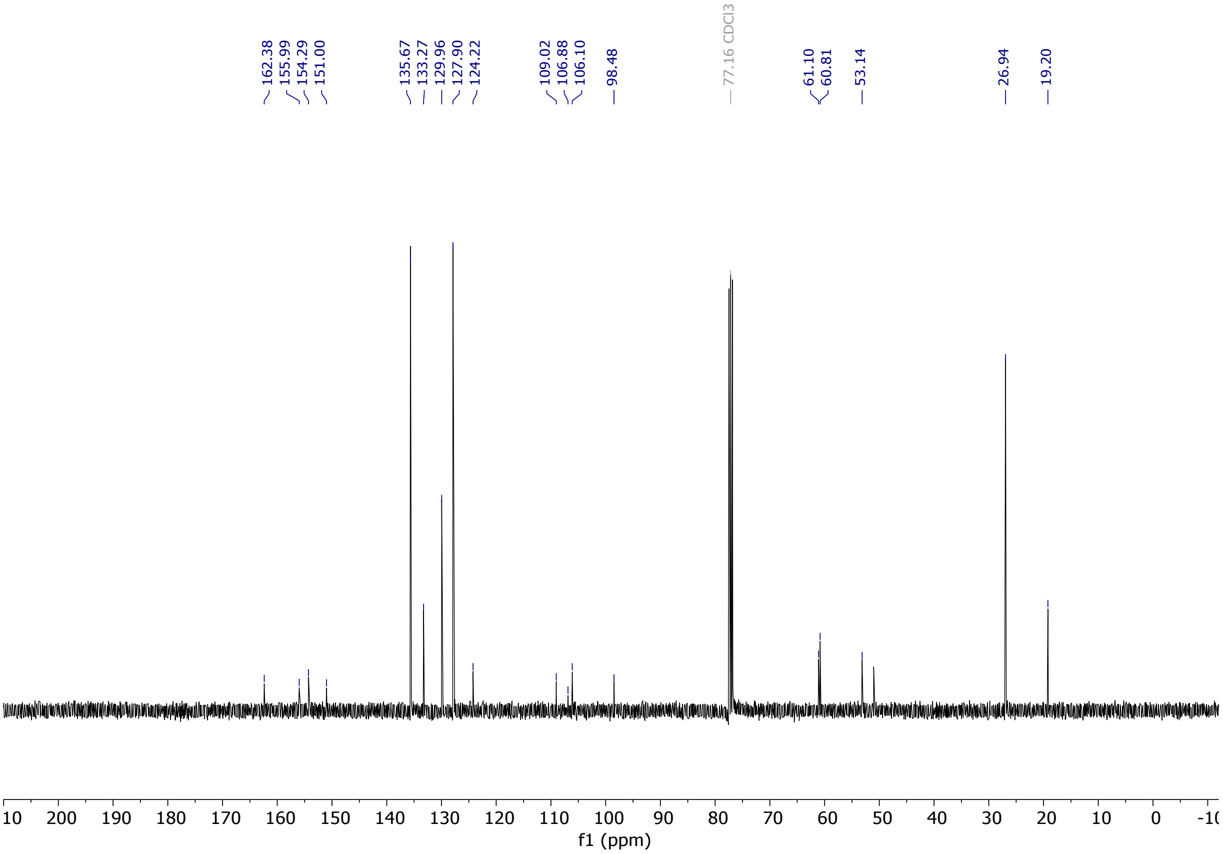

Figure S14. ^{13}C-NMR spectrum of compound **6** ($CDCl_3$)

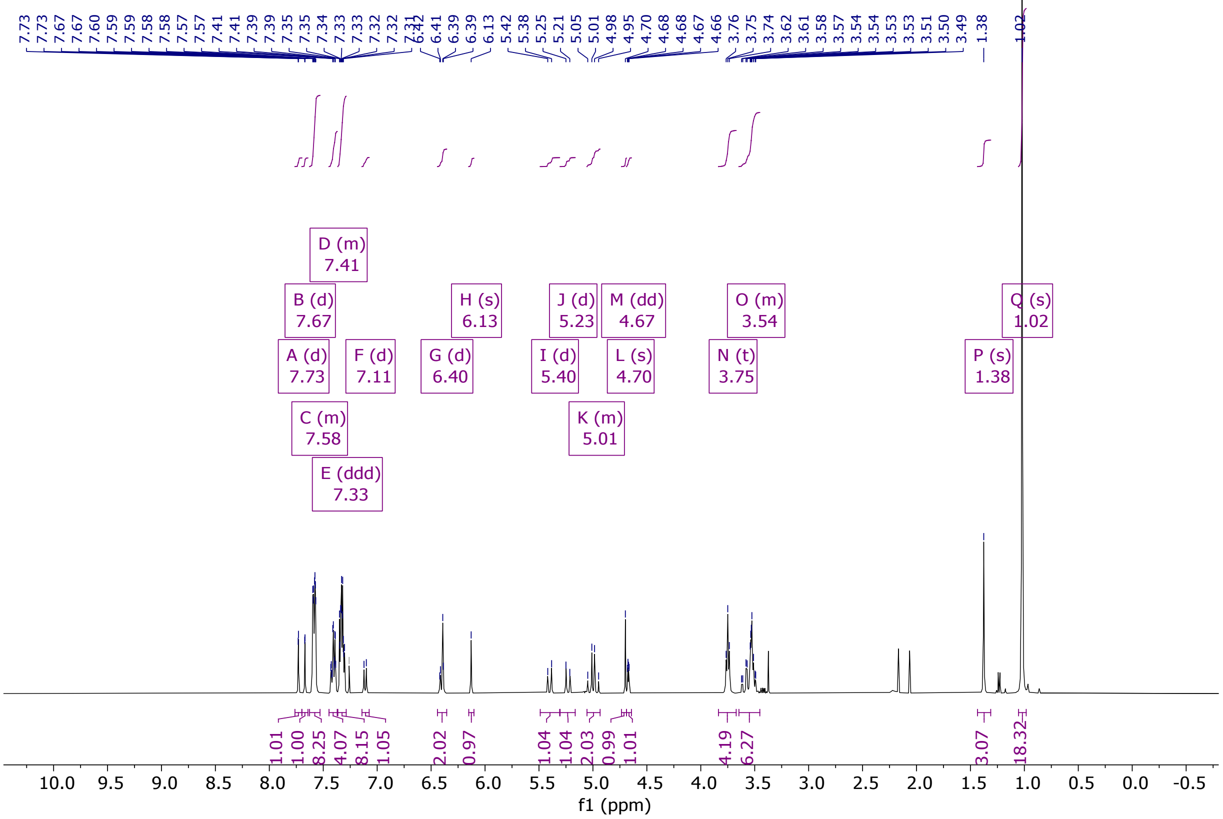

Figure S15. ^{1}H-NMR spectrum of compound **7** ($CDCl_3$)

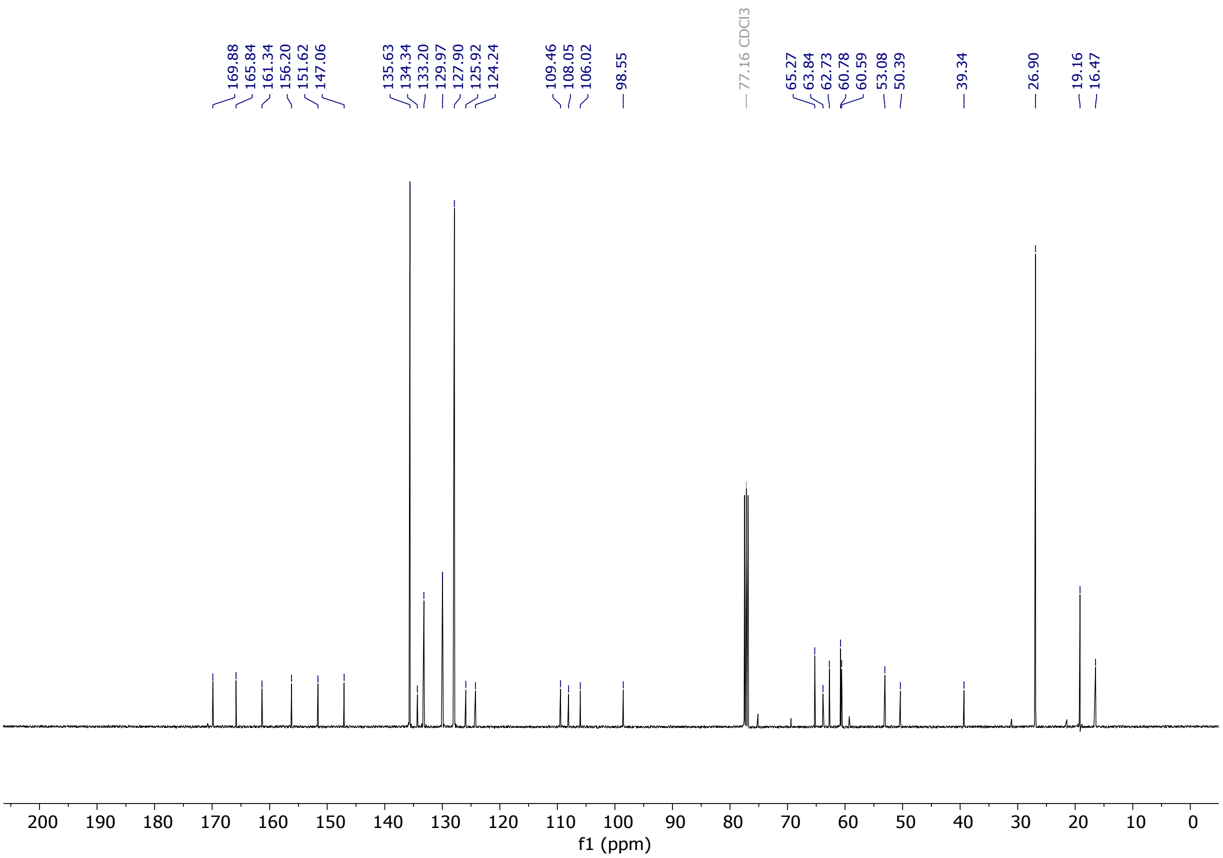

Figure S16. ^{13}C-NMR spectrum of compound **7** ($CDCl_3$)

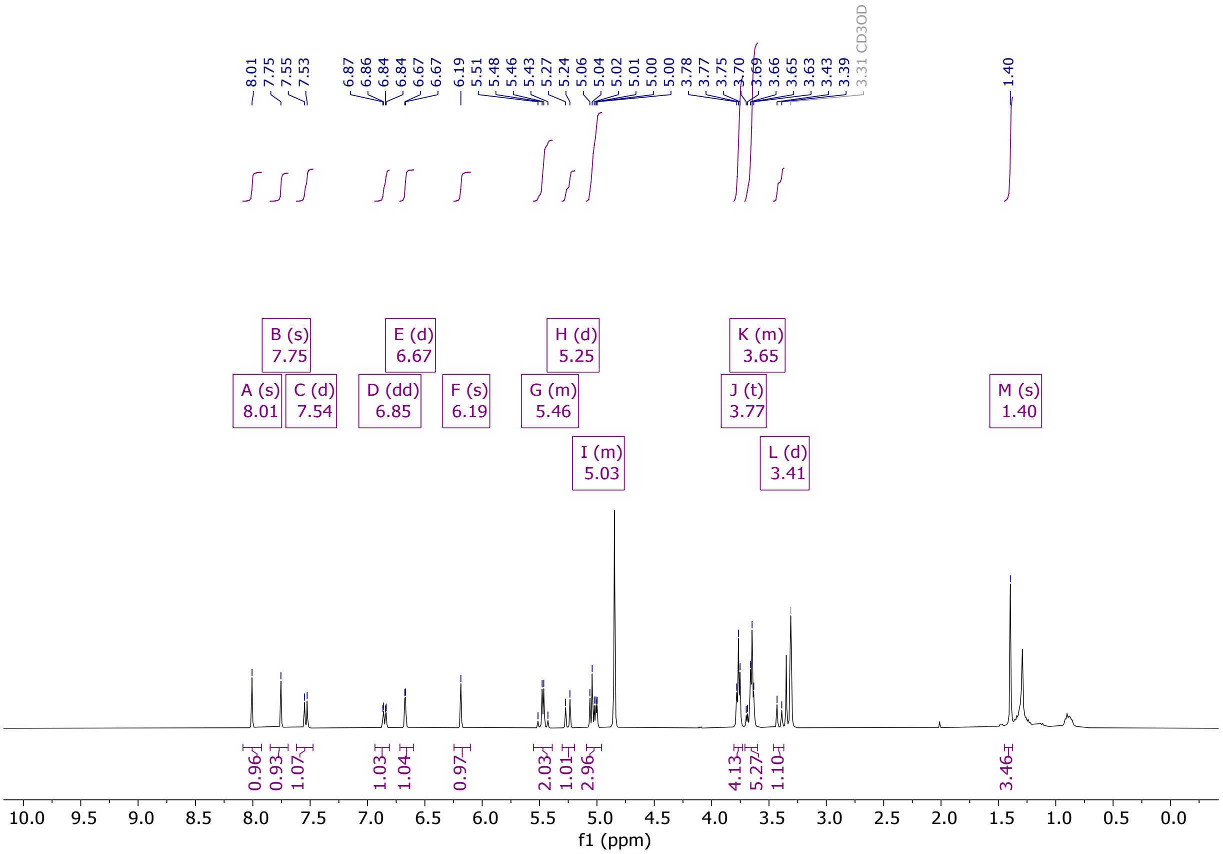

Figure S17. ^{1}H-NMR spectrum of **Tazobactam-PPG** (CD_3OD)

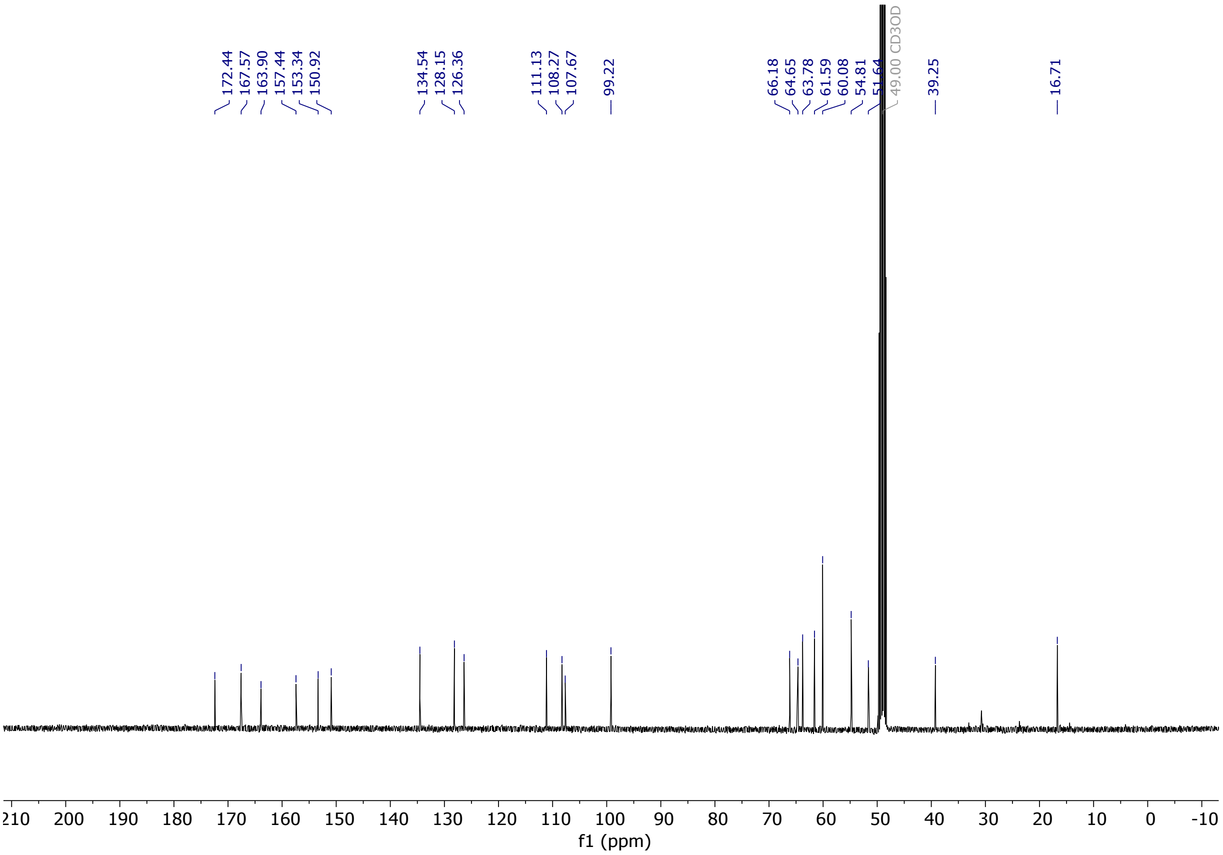

Figure S18. ^{13}C-NMR spectrum of **Tazobactam-PPG** (CD_3OD)

2. Photochemistry

2.1 General

In a typical experiment, a stirred solution of a compound (20 µM, 3 mL) in water with a small amount of organic solvent was irradiated from the side in a fluorescence quartz cuvette (optical path = 1 cm), using a Prizmatix multi-wavelength fiber coupled LED-system (FC10-LED-USB). The full width at half maximum (FWHM) for the 395 nm LED was 12 nm (75 mW, measured photon flux $9.061 \cdot 10^{-5}$ mmol · s^{-1}). The LED was connected through a 7 to 1 fiber bundle (POF 1000 µM) attached to a 3 mm liquid light guide (LLG-3) and a liquid light guide adapter, attached to the cuvette holder of an Agilent Cary 60 UV-Vis spectrophotometer. The LED was controlled automatically via the built-in USB-controller using Prizmatic-Multi-LED-Ctrl & Pulover's MacroCreator. The temperature was maintained at 25 °C or 37 °C using a Cary Single Cell Peltier controller. Raw data was processed using Cary Win-UV, Spectragryph 1.2 and OriginPro 2021.

For irradiation experiments outside of the spectrophotometer, the following LEDs were used:

λ = 400 nm, 3 x Roithner VL-400 Emitter, 3 x 333 mW, FWHM 13 nm.

λ = 530 nm, 3 x LMXL PM01, 810 mW, FWHM 35.1 nm.

λ = 525 nm, 3 x Nichia NCSG219B, 3 x 450 mW, FWHM 32 nm

2.2 ^{1}H-NMR spectra of uncaging of Penicillin-PPG and Tazobactam-PPG

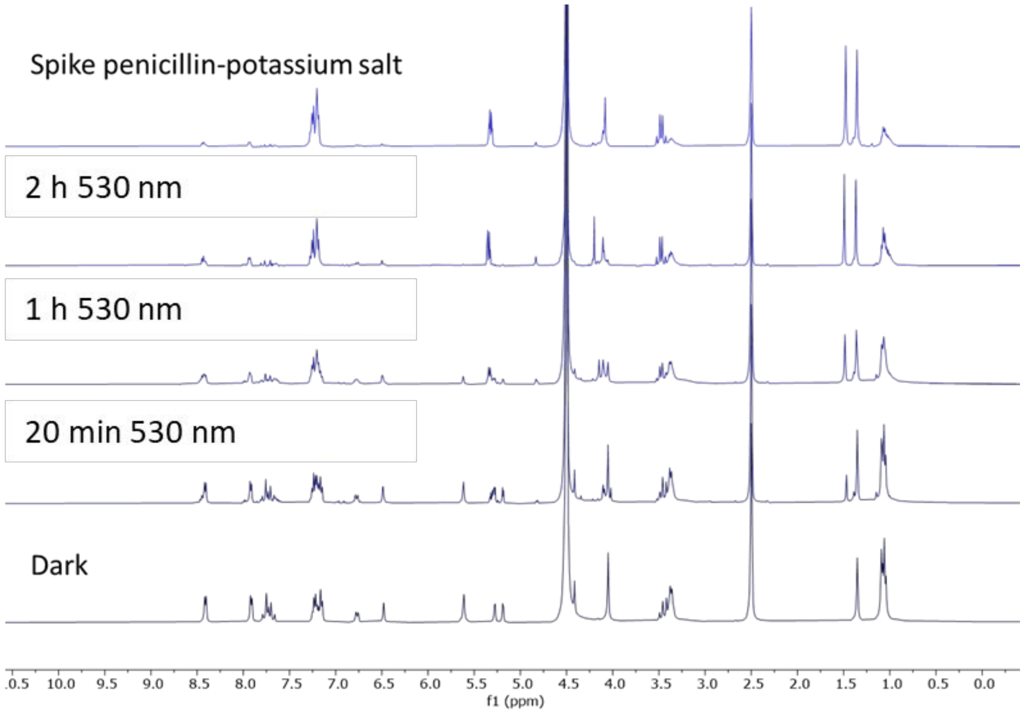

Figure S19. ^{1}H-NMR spectra of **Penicillin-PPG** (2 mM, DMSO-d_6/D_2O 1:1). The bottom spectrum was taken before irradiation, and the top four spectra after irradiation (λ = 530 nm) for different time periods. The addition of benzylpenicillin-potassium salt (top spectrum) confirmed the formation of benzylpenicillin after

irradiation. The observed shift in the signal at 4.2 ppm in the spiked spectrum is due to the addition of a potassium salt.

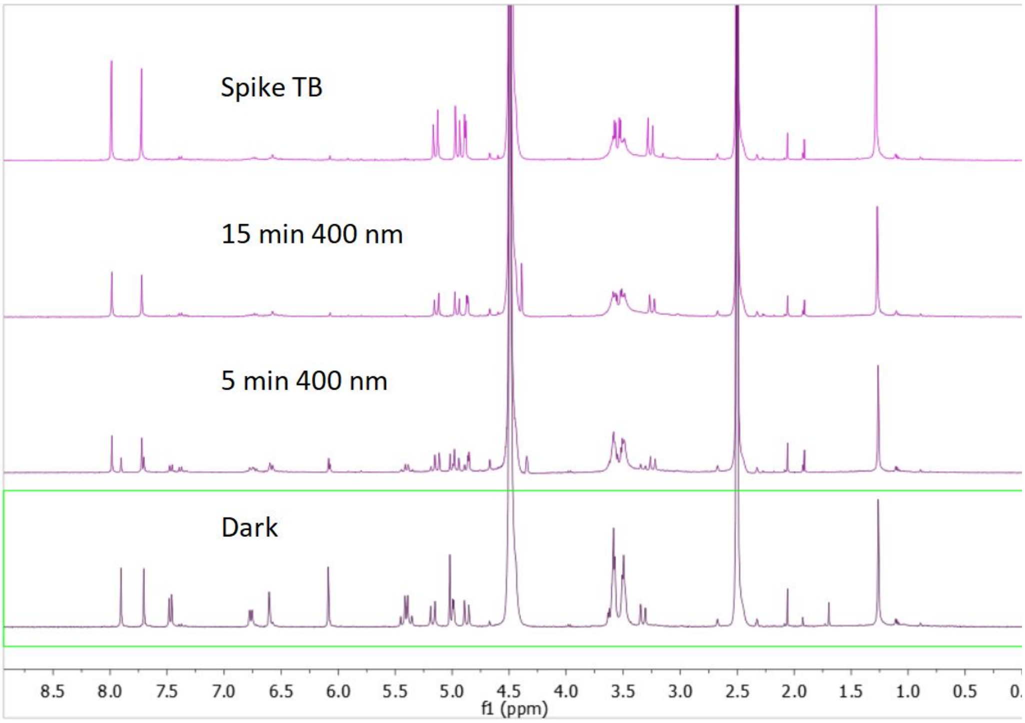

Figure S20. ^{1}H-NMR spectra of **Tazobactam-PPG** (2 mM, DMSO-d_6/D_2O 1:1). The bottom spectrum was taken before irradiation, and the top three spectra after irradiation (λ = 400 nm) for different time periods. The addition of tazobactam (top spectrum) confirmed the formation of tazobactam after irradiation.

2.3 UV-Vis spectra for the uncaging process of Tazobactam-PPG

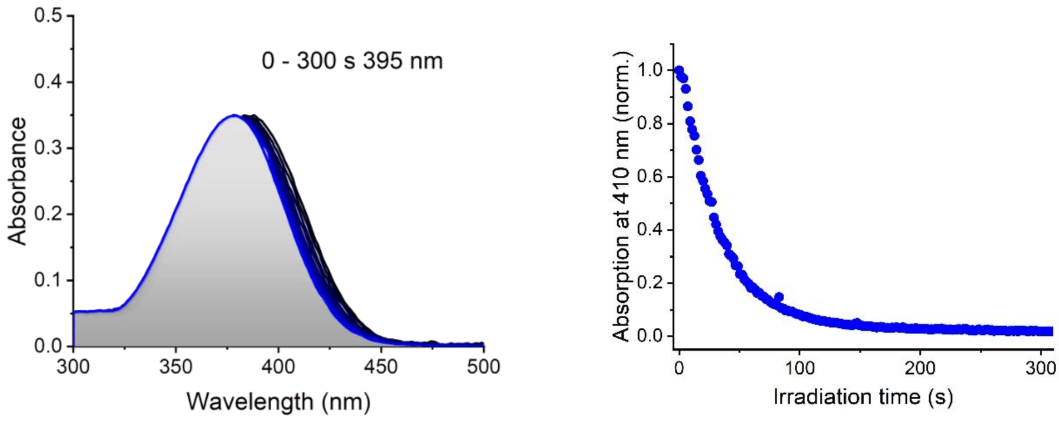

Figure S21. Left) UV-Vis absorption spectra of a sample of **Tazobactam-PPG** (20 µM, water with 0.5 % DMSO, 37 °C) before and after irradiation (λ = 395 nm, black and blue lines respectively, total irradiation time 5 min). Right) Normalized absorption at 410 nm of the spectra of **Tazobactam-PPG** displayed on the left. After 50 s of irradiation, ~ 80 % uncaging was achieved.

2.4 UPLC-MS traces of uncaging Tazobactam-PPG

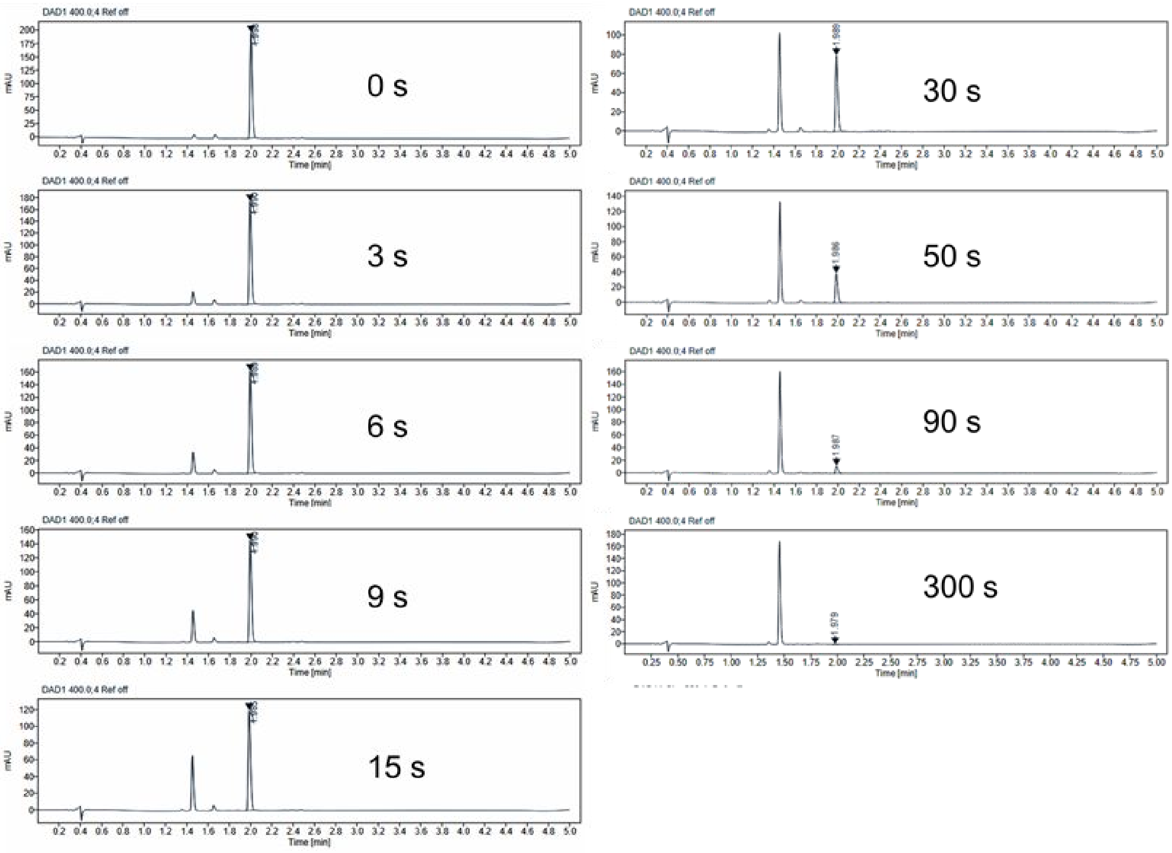

Time (s)	Peak area	RPA
0	295	1.00
3	264	0.89
6	246	0.83
9	221	0.75
15	183	0.62
30	120	0.41
50	59	0.20
90	18	0.06
300	0.3	0.00

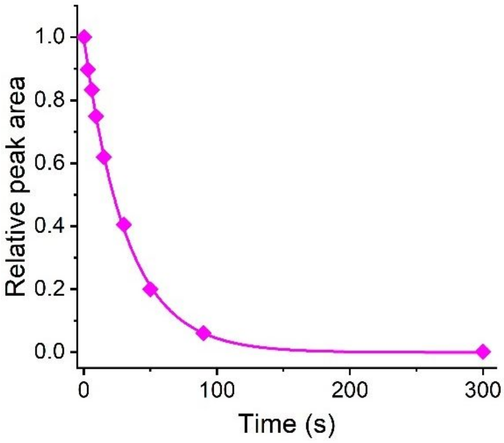

Figure S22. UPLC-MS chromatograms at λ = 400 nm of samples of Tazobactam-PPG (20 µM, water, 0.5 % DMSO) that were irradiated (λ = 395 nm) for the indicated times. For each time point, a fresh solution of Tazobactam-PPG was irradiated for the indicated time. The peak corresponding to Tazobactam-PPG can be seen at 2 min, the formation of the alcohol photoproduct can be observed at 1.5 min. Peak areas were determined through integration of the Tazobactam-PPG peak at 2 min, and the RPA is reported relative to the integral at t 0.

2.5 Molar absorptivity coefficients of Penicillin-PPG and Tazobactam-PPG

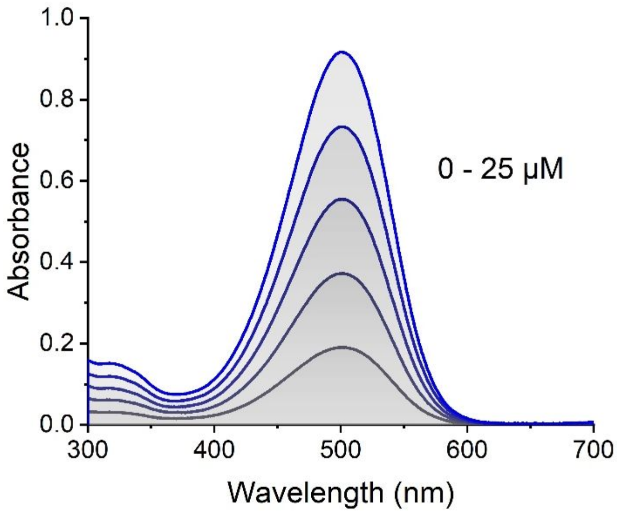

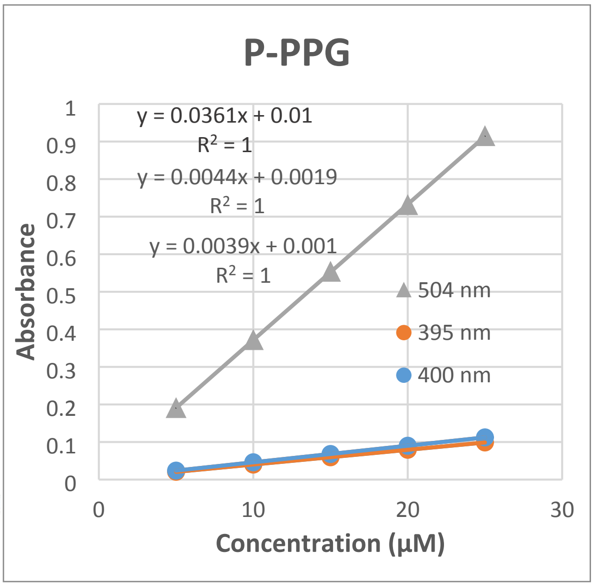

ε at 395 nm : 3910 M^{-1} cm^{-1}

ε at 400 nm : 4420 M^{-1} cm^{-1}

ε at 504 nm (λ_{max}): 36100 M^{-1} cm^{-1}

Figure S23. UV-Vis absorption spectra of Penicillin-PPG (5 – 25 μM, 0.1 – 0.6 % DMSO in water, 37 °C) and molar absorptivity values at irradiation wavelengths and at λ_{max}.

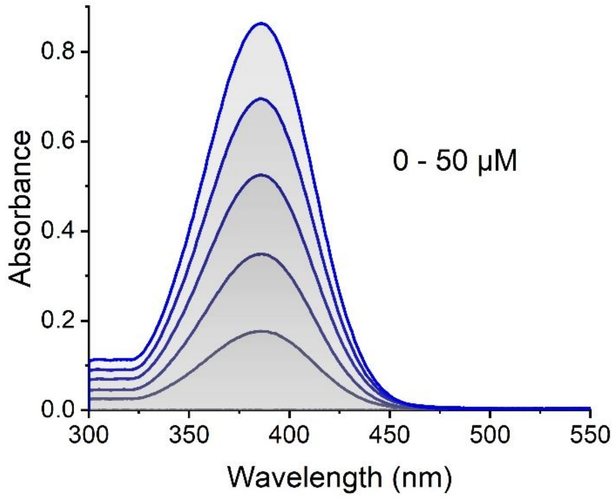

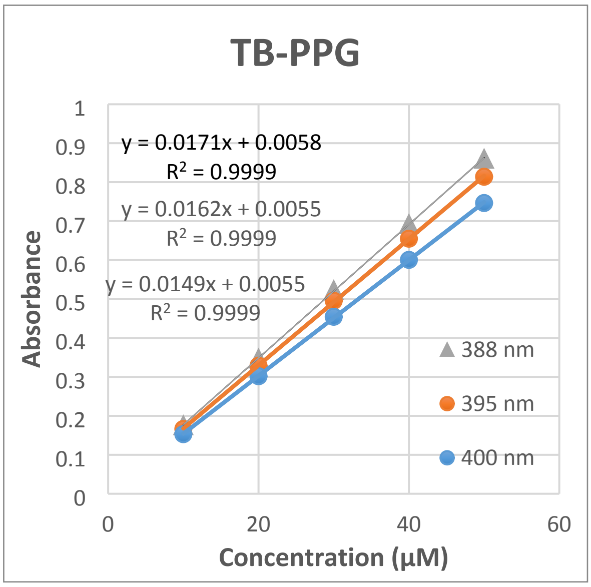

ε at 395 nm : 16200 M^{-1} cm^{-1}

ε at 400 nm : 14900 M^{-1} cm^{-1}

ε at 388 nm (λ_{max}): 17100 M^{-1} cm^{-1}

Figure S24. UV-Vis absorption spectra of Tazobactam-PPG (10 – 50 μM, 0.3 - 1.3 % DMSO in water, 37 °C) and molar absorptivity values at irradiation wavelengths and at λ_{max}.

2.6 UPLC-MS traces of uncaging of Penicillin-PPG and Tazobactam-PPG

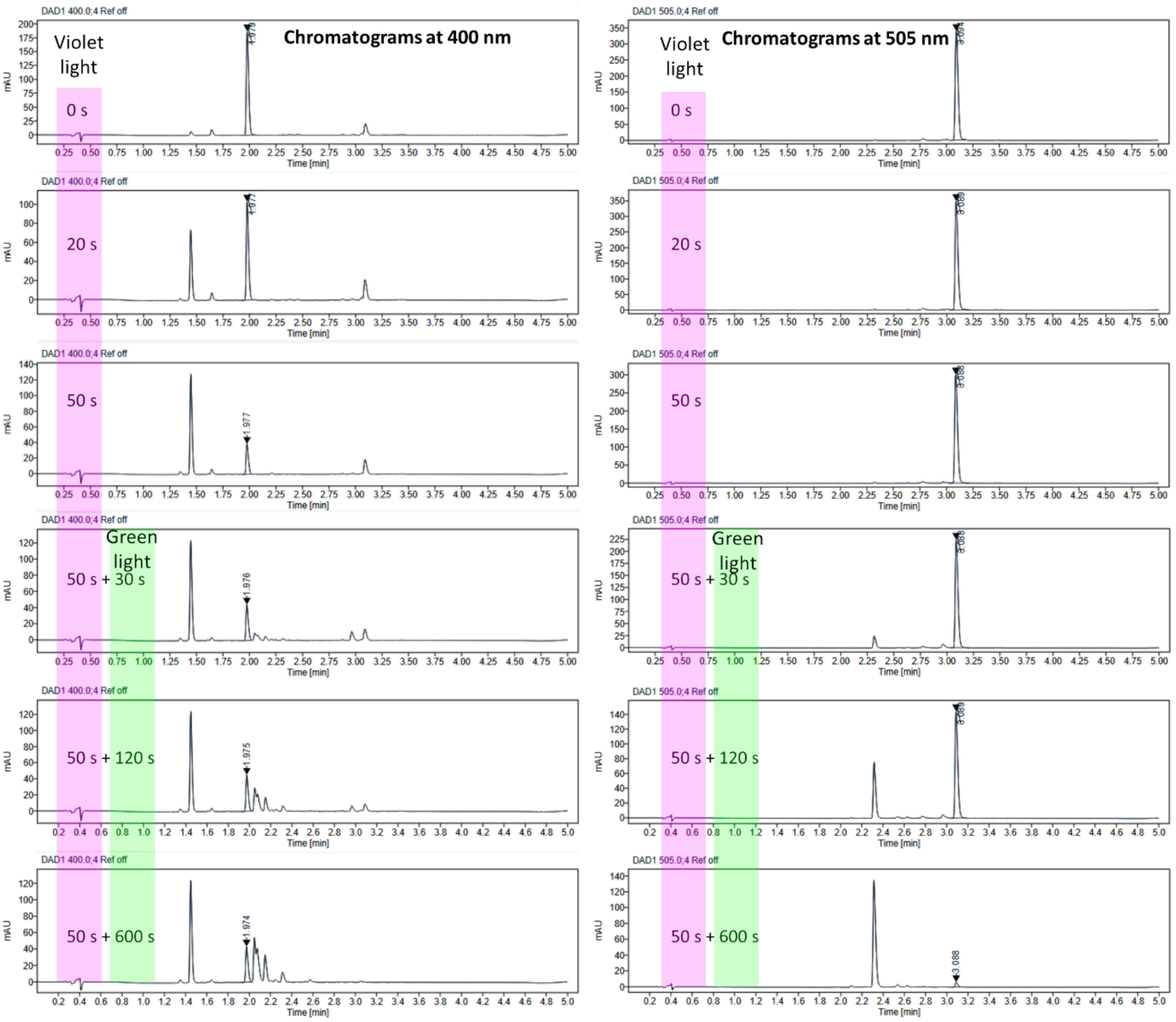

Figure S25. UPLC-MS chromatograms at λ = 400 nm (left) and λ = 505 nm (right) of samples containing a mixture of Tazobactam-PPG and Penicillin-PPG (both 20 µM, 99:1 water/DMSO, 3 mL, 37 °C) that were irradiated (violet light, λ = 395 nm and green light, λ = 525 nm) for the indicated total irradiation times. Solutions were irradiated and 100 µL samples were taken for UPLC-MS analysis. To avoid errors based on concentration differences, at most two samples were taken from the same experiment, after which a fresh solution was prepared. The peak corresponding to Tazobactam-PPG can be seen at 2 min in the chromatograms at λ = 400 nm, the peak corresponding to Penicillin-PPG can be seen at 3.1 min in the chromatograms at λ = 505 nm.

Tazobactam-PPG

	Peak areas			Relative peak areas				
	#1	#2	#3	#1	#2	#3	Average RPA	SD
**t 0**	292	280	255	1.06	1.02	0.93	1.00	0.06
**20 s V**	144	157	159	0.52	0.57	0.58	0.56	0.02
**50 s V**	66	70	60	0.24	0.25	0.22	0.24	0.01
**50 s V + 30 s G**	70	73	68	0.25	0.26	0.25	0.26	0.01
**50 s V + 120 s G**	70	67	66	0.25	0.24	0.24	0.25	0.01
**50 s V + 600 s G**	66	66	68	0.24	0.24	0.25	0.24	0.00

Table S1. Peak areas and relative peak areas of the peak corresponding to Tazobactam-PPG in the chromatogram at λ = 400 nm. The relative peak areas were determined by dividing each peak area by the average peak area at t 0 (276).

Penicillin-PPG

	Peak areas			Relative peak areas				
	#1	#2	#3	#1	#2	#3	Average RPA	SD
**t 0**	634	667	511	1.05	1.10	0.85	1.00	0.11
**20 s V**	517	655	691	0.86	1.08	1.14	1.03	0.12
**50 s V**	692	563	565	1.15	0.93	0.94	1.00	0.10
**50 s V + 30 s G**	478	493	419	0.79	0.82	0.69	0.77	0.05
**50 s V + 120 s G**	256	251	200	0.42	0.42	0.33	0.39	0.04
**50 s V + 600 s G**	10	12	13	0.02	0.02	0.02	0.02	0.00

Table S2. Peak areas and relative peak areas of the peak corresponding to Penicillin-PPG in the chromatogram at λ = 505 nm. The relative peak areas were determined by dividing each peak area by the average peak area at t 0 (604).

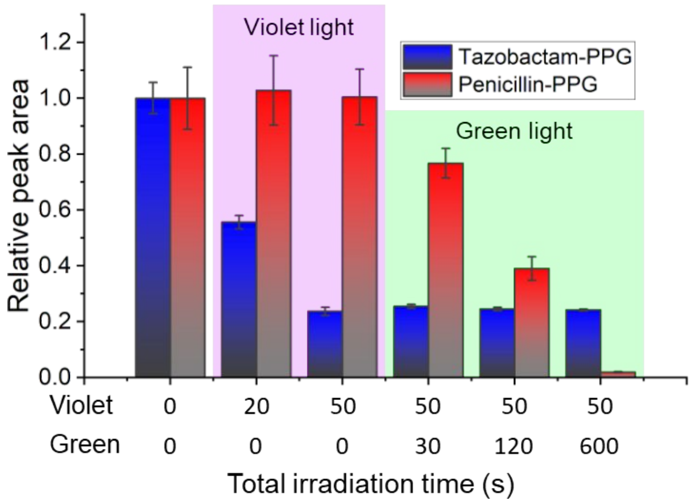

Figure S26. Plotted relative peak areas versus irradiation time, data from table S1 and table S2.

3. Biological experiments

3.1 MIC-value determination

3.1.1 Benzylpenicillin towards *E. coli* DH5α

Stock solutions of benzylpenicillin potassium salt in DMSO (ranging from 9.5 - 6 mM) were diluted with Lysogeny Broth (LB; 50 x) and aliquoted (3 x 100 µL per concentration) in a clear, sterile 96-well plate (final 2 % DMSO in all samples). Subsequently, the samples were inoculated with an overnight culture of *E. coli* DH5α grown in LB (100 µL, diluted to OD 0.002). The plate was placed in a Biotek Synergy H1 plate-reader, and incubated for 24 h at 37 °C. The growth curves were recorded by following the optical density at 650 nm (OD_{650}) every 10 min with a 10 s shaking step before each measurement.

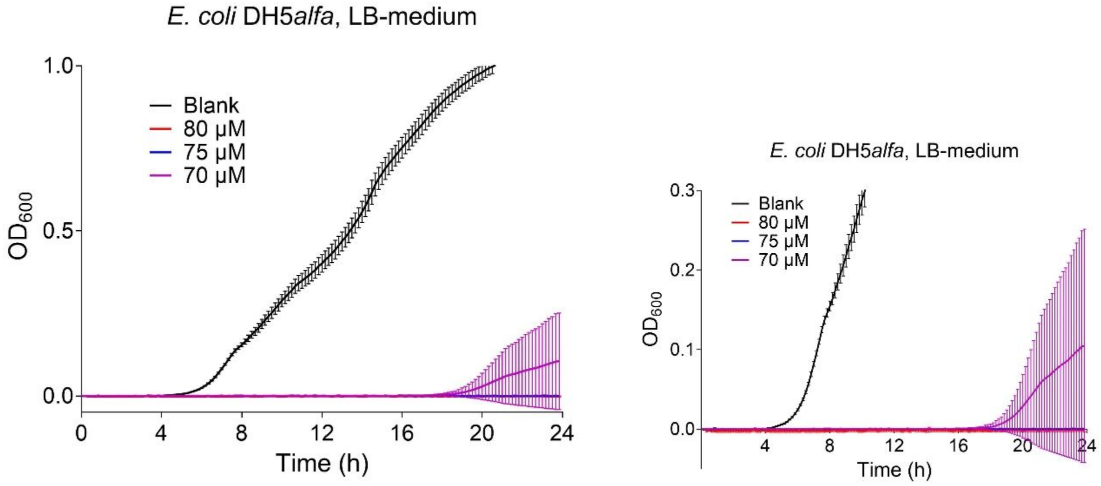

Figure S27. MIC-value determination for benzylpenicillin-potassium salt towards E. coli DH5α. Reported are averages and SD-values of triplicate measurements. A zoomed in version of the growth curves is displayed on the right.

3.1.2 Benzylpenicillin and tazobactam towards *E. coli* DH5α + PET vector

Stock solutions of benzylpenicillin potassium salt and tazobactam in DMSO were diluted with LB to prepare a range of samples containing both compounds at different concentrations (with the final DMSO content of 2 % in all the samples). The samples were aliquoted (3 x 100 µL per concentration) in a clear, sterile 96-well plate. Subsequently, they were inoculated with an overnight culture of *E. coli* DH5α + PET vector, grown in LB (100 µL, diluted to OD 0.002). The plate was placed in a Biotek Synergy H1 plate-reader, and incubated for 24 hours at 37 °C. The growth curves were recorded by following the optical density at 650 nm (OD_{650}) every 10 min with a 10 s shaking step before each measurement.

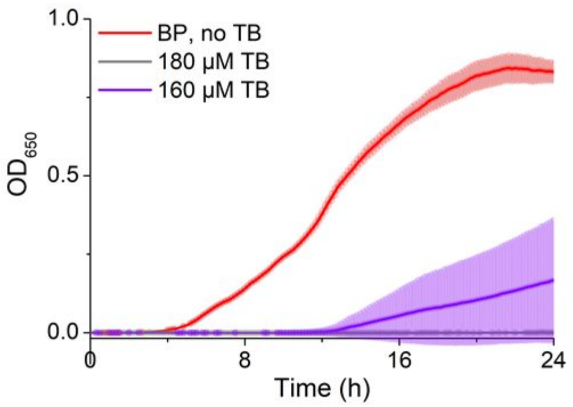

Figure S28. MIC-value determination for benzylpenicillin and tazobactam towards *E. coli* DH5α + PET vector. Reported are averages and SD-values of triplicate measurements. A concentration of 80 µM benzylpenicillin was used in each sample, and concentrations of tazobactam (TB) were varied.

3.2 Light dependent inhibition of bacterial growth in liquid culture

3.2.1 Penicillin-PPG – *E. coli* DH5α

Stock solutions of **Penicillin-PPG** (48 µL, 2.67 mM, DMSO) and **OAc-PPG** (48 µL, 2.67 mM, DMSO) were diluted in sterile LB (752 µL LB). A 'blank' sample was prepared by diluting 48 µL DMSO with 752 µL LB. For each mixture, 3 x 100 µL was aliquoted in a sterile 96-well plate, and the rest was kept at room temperature in a sterile dark Eppendorf tube. The 96-well plate containing the samples was irradiated with green light (λ = 530 nm) for 1 h. Subsequently, for each mixture another three aliquots (100 µL) were added to the 96-well plate ('dark' samples). All samples were inoculated with an overnight culture of *E. coli* DH5α (100 µL, diluted to OD 0.002) and the plate was placed in a Biotek Synergy H1 plate-reader and incubated for 24 h at 37 °C. The growth curves were recorded by following the optical density at 650 nm (OD_{650}) every 10 min with a 10 s shaking step before each measurement.

3.2.2 Penicillin-PPG and Tazobactam-PPG – *E. coli* DH5α + PET-vector

A DMSO solution containing **Penicillin-PPG** and **Tazobactam-PPG** (96 µL, 3.00 mM and 6.67 mM respectively) was diluted in sterile LB (1.50 mL LB) to create a master mix. A 'blank' sample was prepared by diluting 48 µL DMSO with 752 µL LB and part of it was aliquoted (3 x 100 µL) in a sterile 96-well plate. Part of the master mix was also aliquoted (2 times triplicates, 100 µL) in the 96-well plate, and the rest of the mixture was kept at room temperature in a sterile dark Eppendorf tube. The plate was irradiated with violet light (λ = 400 nm) for 1 min. Subsequently, the samples that were not to be irradiated with green light (3 * 100 µL) were transferred back into a separate, sterile dark Eppendorf tube labelled 'violet'. An additional 3 * 100 µL of the master mix was aliquoted into the 96-well plate, that was subsequently irradiated with green light (λ = 530 nm) for 1 h. Afterwards, the 'violet' samples were transferred back to the plate, as well as another 3 aliquots (100 µL) of the master mix ('dark' samples) and another 3 aliquots (100 µL) of the blank sample (DMSO, dark). All samples were inoculated with an overnight culture of *E. coli* DH5α + PET-vector (100 µL, diluted to OD_{650} 0.002) and the plate was placed in a Biotek Synergy H1 plate-reader and incubated for 24 hours at 37 °C. The growth curves were recorded by following the optical density at 650 nm (OD_{650}) every 10 min with a 10 s shaking step before each measurement.

3.3 Petri-dish experiments

3.3.1 Penicillin-PPG – *E. coli* DH5α

LB-Agar (15 g/L agar) was autoclaved and cooled until hand-warm. 11.6 mL was aliquoted three times in sterile Greiner tubes. To the tubes were added stock solutions of either **Penicillin-PPG** (360 µL, 3 mM in DMSO), benzylpenicillin-potassium salt (360 µL, 3 mM in DMSO) or DMSO (360 µL). After gentle mixing, the 3 mixtures were poured into 3 separate sterile petri-dishes and left to solidify. After solidification, the plates were inoculated with a liquid sample of an overnight culture of *E. coli* DH5α (400 µL, diluted to OD 0.002) and the mixture was spread out over the plate aseptically until all was absorbed. Subsequently, half of the bottom of the petri dishes was covered with an aluminium sticker, and the uncovered half was irradiated with green light for 1 hour (λ = 530 nm). After irradiation, the plates were incubated overnight at 37 °C.

3.3.2 Penicillin-PPG and Tazobactam-PPG – *E. coli* DH5α + PET-vector

LB-Agar (15 g/L agar) was autoclaved and cooled until hand-warm. 11.6 mL was aliquoted 2 times in sterile Greiner tubes. To one of the tubes was added stock solutions of **Penicillin-PPG** (180 µL, 5.33 mM in DMSO) and **Tazobactam-PPG** (180 µL, 12 mM in DMSO). To another tube was added benzylpenicillin-potassium salt (180 µL, 5.33 mM in DMSO). After gentle mixing, the 2 mixtures were poured into 2 separate sterile petri-dishes and left to solidify. After solidification, the plates were inoculated with a liquid sample of an overnight culture of *E. coli* DH5α + PET vector (400 µL, diluted to OD 0.002) and the mixture was spread out over the plate aseptically until all was absorbed. Subsequently, the lid and bottom of the petri dishes were covered with an aluminium sticker such that a middle slit was created. The petri dish was placed in a clamp, and irradiated through the slit on the lid with green light (λ = 530 nm) for 1 h, and through the slit on the bottom with violet light (λ = 400 nm) for 30 s. The plates were incubated overnight at 37 °C.

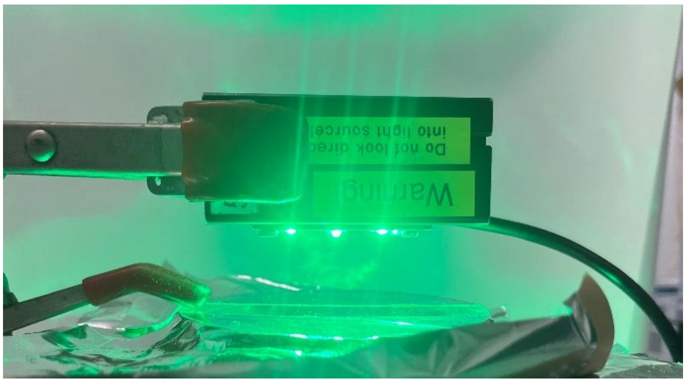

Figure S29. Irradiation of the partly covered petri dish (bottom) with the green LED.

3.4 Prevention of biofilm formation using Penicillin-PPG

The MIC-value of the *S. epidermidis* ATCC 35984 strain was determined as described above (section 3.1), with the exception that Tryptic Soy Broth (TSB) was used as growth medium. Moreover, because of a different set-up in a different laboratory, a starting OD of 0.05 was chosen, the OD was measured at 600 nm using a Biotek Synergy 2.0 microplate reader (BioTek Instruments, Inc., USA), and measurements were performed every 15 min.

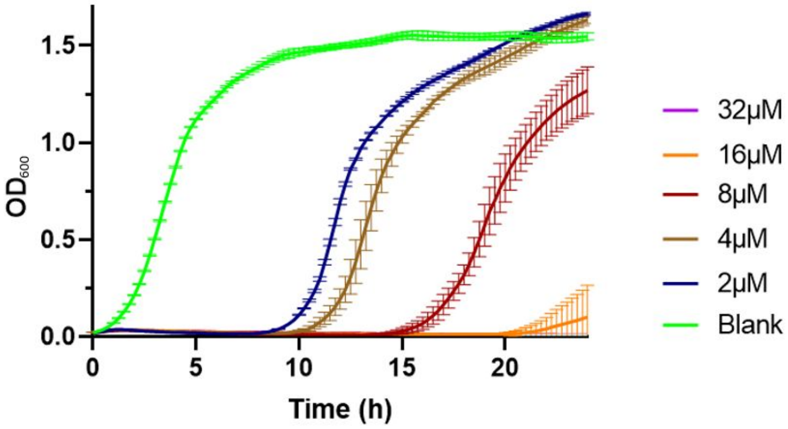

Figure S30. Minimum inhibitory concentration determination for benzylpenicillin-potassium salt towards the *S. epidermidis* American Type Culture Collection (ATCC) 35984 strain. Reported are averages and SD-values of triplicate measurements.

For the biofilm experiments, an *S. epidermidis* overnight culture was diluted 1:50 in fresh TSB and incubated for 2 h at 37 °C in a shaking incubator. When the bacteria reached exponential growth, the bacterial culture was diluted to an OD_{600} of 0.05 in TSB supplemented with 5% glucose and 4% sodium chloride in the presence of sterile coverslips. To the coverslips were added (n = 8 per group): 52 µM benzylpenicillin (final 2% DMSO), 52 µM **Penicillin-PPG** (final 2% DMSO), or 2% DMSO without antibiotic. Then, half of the coverslips of each group were irradiated with green light (λ = 530 nm) for 2 h, whereas the other coverslips of each group were kept in the dark. After irradiation, all coverslips were placed in a dark incubator at 37 °C and biofilms were allowed to form on the coverslips over 24 h.

For biofilm staining, two coverslips per group (12 in total) were washed with phosphate-buffered saline (PBS) to remove all planktonic bacteria, and subsequently submerged in a Crystal Violet solution (0.2%) for 15 min. The coverslips were then washed 5 times with PBS to remove the unbound stain. White-light images of the biofilms were captured.

To determine colony-forming units (CFU), two coverslips per group (12 in total) were washes with PBS to remove all planktonic bacteria. Each coverslip was placed in a sterile container with 25 mL Ringer Lactate and sonicated for 3 min, whereafter sterile dilutions of the sonication-fluid were made and 10 µL of each fluid was plated on blood agar plates (5% sheep blood, Mediaproducts B.V., the Netherlands). CFUs were counted after 24 h growth at 37 °C.

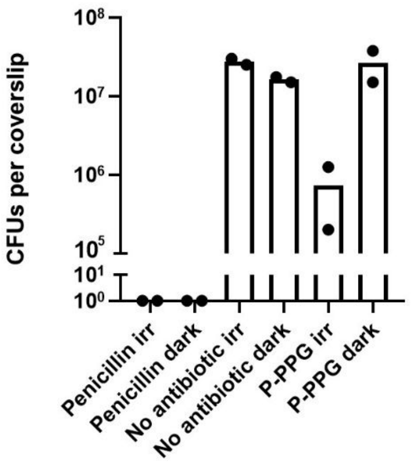

Figure S31. CFUs of *S. epidermidis* ATCC 35984 present in biofilms on coverslips, displayed on a logarithmic scale. Biofilms were grown in 24 h on glass coverslips after 2 h green light irradiation or no irradiation (dark) in the presence of **Penicillin-PPG**, benzylpenicillin, or no antibiotic. After 24 h, coverslips were washed to remove planktonic bacteria and sonicated for 3 min, whereafter dilutions of the sonication-fluid were made and plated on blood agar plates.

3.5 In vivo treatment of bacterial infection with green light-activated Penicillin-PPG

The MIC-value of the *S. aureus* SH1000 strain was determined as described for the biofilm experiments.

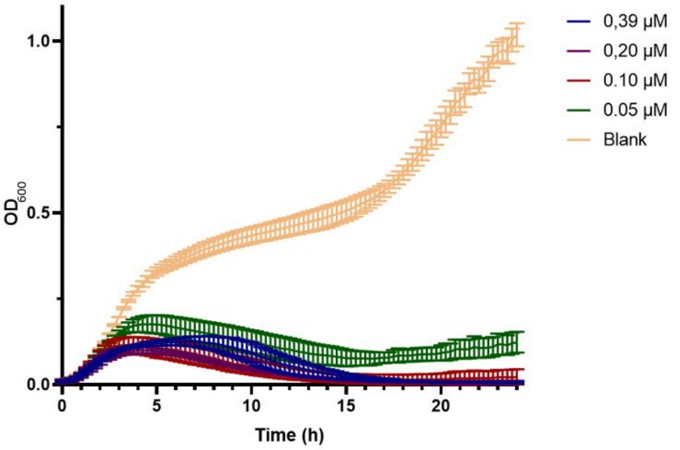

Figure S32. Minimum inhibitory concentration determination for benzylpenicillin-potassium salt towards the *S. aureus* SH1000 strain.

G. mellonella larvae (Frits Kuiper, Groningen, the Netherlands) in their final instar stage were purchased and weighed. Larvae between 250-300 mg that showed no signs of melanisation (which is a high predictor of larval mortality[4]) were selected and randomized between groups. Each group consisted of 30 larvae and all experiments were performed twice. Control larvae were either not injected or injected in the hemocoel of the last proleg using an insulin pen (HumaPen LUXURA® HD, Indianapolis, USA) with 10 µL PBS or 10 µL **Penicillin-PPG** (0.4 µM, 2% DMSO in PBS). Experimental larvae were injected in the hemocoel of the last proleg with 10 µL 0.5 x 10^6 CFUs of *S. aureus* SH1000, harvested during exponential growth and dissolved in PBS, or 10 µL 0.5 x 10^6 CFUs of *S. aureus* SH1000

in exponential growth and supplemented with **Penicillin-PPG** (0.4 µM, 2% DMSO in PBS). Half of the larvae were subjected to 2 h irradiation with green light (λ = 530 nm) and the other half were kept in the dark. Then, all larvae were incubated in the dark in petri dishes at 37 °C for 4 days. Mortality was monitored at 0, 24, 48, 72 and 96 h post irradiation. Complete melanisation plus irresponsiveness to touch were used as the criterion for larval death.[5] Kaplan-Meier survival curves, Gehan-Breslow-Wilxocon tests and two-way ANOVA with subsequent Sidak multiple comparisons tests were calculated in GraphPad Prism 8.1.1 (GraphPad Software, CA, USA). A p-value of <0.05 was considered significant.

4. References

1. Bojtár, M., Kormos, A., Kis-Petik, K., Kellermayer, M. & Kele, P. Green-Light Activatable, Water-Soluble Red-Shifted Coumarin Photocages. *Org Lett* **21**, 9410–9414 (2019).
2. Huang, Q., Bao, C., Ji, W., Wang, Q. & Zhu, L. Photocleavable coumarin crosslinkers based polystyrene microgels: Phototriggered swelling and release. *J Mater Chem* **22**, 18275–18282 (2012).
3. Shah, S. T. A. & Guiry, P. J. The chemoselective and efficient deprotection of silyl ethers using trimethylsilyl bromide. *Org Biomol Chem* **6**, 2168–2172 (2008).
4. Tsai, C. J.-Y., Loh, J. M. S. & Proft, T. Galleria mellonella infection models for the study of bacterial diseases and for antimicrobial drug testing. *Virulence* **7**, 214–29 (2016).
5. Loh, J. M., Adenwalla, N., Wiles, S. & Proft, T. *Galleria mellonella* larvae as an infection model for group A streptococcus. *Virulence* **4**, 419–428 (2013).
6. Blue laserpointer - Laserpointerpro. https://www.laserpointerpro.com/5mw-405nm-beam-light-purple-laser-pointer-pen-p-942.html.
7. FOBSERD ZK-9318 - Amazon. https://www.amazon.com/Presentation-Construction-Measurement-Instructions-Flashlight/dp/B099F7XF2Q.
